# The Translational Potential of Microglia and Monocyte-Derived Macrophages in Ischemic Stroke

**DOI:** 10.3389/fimmu.2022.897022

**Published:** 2022-06-20

**Authors:** Elizabeth E. Wicks, Kathleen R. Ran, Jennifer E. Kim, Risheng Xu, Ryan P. Lee, Christopher M. Jackson

**Affiliations:** Department of Neurosurgery, The Johns Hopkins University School of Medicine, Baltimore, MD, United States

**Keywords:** microglia, monocyte-derived macrophages, ischemic stroke, polarization, clinical therapy/immunology, immune response, acute/subacute ischemic stroke, chronic ischemic stroke

## Abstract

The immune response to ischemic stroke is an area of study that is at the forefront of stroke research and presents promising new avenues for treatment development. Upon cerebral vessel occlusion, the innate immune system is activated by danger-associated molecular signals from stressed and dying neurons. Microglia, an immune cell population within the central nervous system which phagocytose cell debris and modulate the immune response *via* cytokine signaling, are the first cell population to become activated. Soon after, monocytes arrive from the peripheral immune system, differentiate into macrophages, and further aid in the immune response. Upon activation, both microglia and monocyte-derived macrophages are capable of polarizing into phenotypes which can either promote or attenuate the inflammatory response. Phenotypes which promote the inflammatory response are hypothesized to increase neuronal damage and impair recovery of neuronal function during the later phases of ischemic stroke. Therefore, modulating neuroimmune cells to adopt an anti-inflammatory response post ischemic stroke is an area of current research interest and potential treatment development. In this review, we outline the biology of microglia and monocyte-derived macrophages, further explain their roles in the acute, subacute, and chronic stages of ischemic stroke, and highlight current treatment development efforts which target these cells in the context of ischemic stroke.

## Introduction

First described by Hippocrates nearly 2,400 years ago, stroke, or “apoplexy,” is the second leading cause of death globally and accounts for approximately 1 out of every 19 deaths in the United States (1). Stroke is also a leading cause of long-term disability and places a high economic burden on global healthcare systems. Despite significant advances in primary and secondary stroke prevention, the annual number of strokes and stroke-related deaths have persistently increased over the past two decades (2). An estimated 87% of strokes are ischemic (3), in which a sudden interruption in cerebral blood flow results in rapid cell death within the ischemic core. Specifically in the neuropathological progression of stroke, neuronal necrosis and apoptosis result in profound neuroinflammation and secondary tissue injury, which can be counterproductive to both short and long-term recovery (4, 5).

Current treatment strategies for acute thrombotic or embolic stroke are focused on early reperfusion with intravenous thrombolytics or mechanical thrombectomy, supplemented by supportive care and acute complication management. At present, alteplase is the only Food and Drug Administration (FDA)-approved medical therapy in the United States for the treatment of acute ischemic stroke (6). Mechanical thrombectomy with or without intravenous thrombolysis has revolutionized the treatment of stroke. Similar progress in treating secondary inflammatory injury is necessary to optimize patient outcomes.

### The Immune Response to Stroke

Inflammation plays a critical role in the pathogenesis of ischemic stroke. Hypoxic brain injury results in rapid activation of resident immune cells and subsequent influx of peripheral inflammatory cells (7). The innate immune response occurs in three phases following an ischemic insult: the acute stage (minutes to hours), the subacute stage (hours to days), and the chronic stage (days to months) (8). In each of these phases, specific immune cell populations participate in tissue repair; however, aberrant, overly robust, or prolonged inflammation at any stage can be counterproductive to recovery. Elucidating the specific cell types active at each stage, their roles in tissue repair, and how they interact within the neurovascular unit is critical to developing immune-based therapies to mitigate secondary injury.

**Figure 1:**
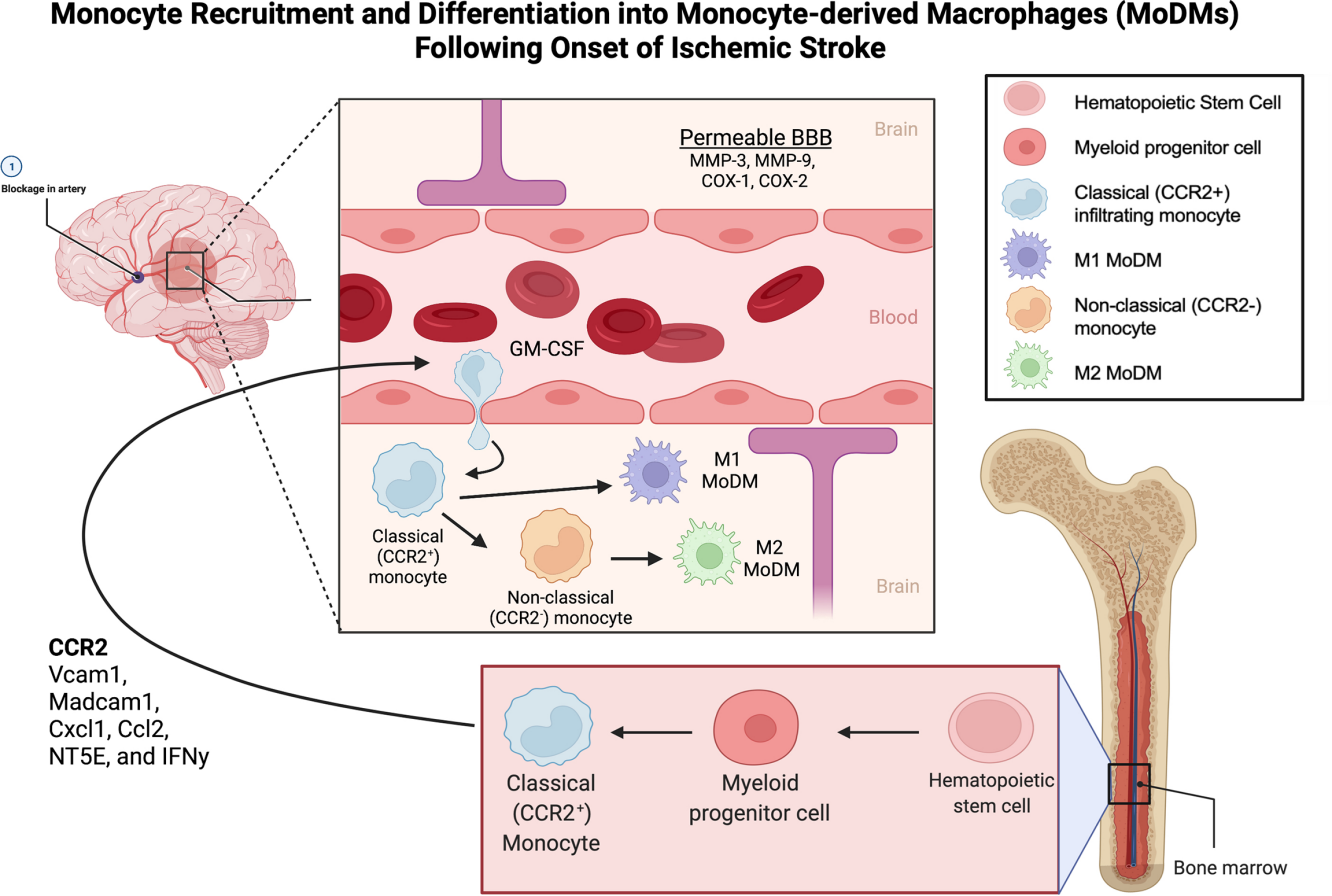
Monocyte Recruitment and Differentiation into Monocyte-derived Macrophages (MoDMs) Following Onset of Ischemic Stroke. Monocytes originate from myeloid progenitor cells derived from hematopoietic stem cells in the bone marrow. Upon ischemic insult, the classical monocytes (CCR2+) are recruited to the area of inflammation through the release of CCR2, Vcam1, Madcam1, Cxcl1, Ccl2, NT5E, and IFNy. MMP-3, MMP-9, COX-1 and COX-2 facilitate the breakdown of the BBB allowing extravasation of the classical monocytes into the brain parenchyma. Monocyte migration is enhanced by GM-CSF. On arriving to the ischemic tissue, monocytes differentiate into MoDMs in response to chemokines, interleukins, and granule proteins produced by microglia, astrocytes, and neutrophils. In the ischemic site, classical monocytes can also lose expression of CCR2 to assume the non-classical phenotype. Classical monocytes primarily differentiate into M1, pro-inflammatory MoDMs while non-classical monocytes primarily differentiate into M2 anti-inflammatory MoDMs. MoDM, monocyte derived macrophage; GM-CSF, Granulocyte-macrophage colony-stimulating factor; BBB, Blood brain barrier; CCR2, C-C Motif Chemokine Receptor 2; Vcam1, Vascular Cell Adhesion Molecule-1; Madcam1, Mucosal Vascular Addressin Cell Adhesion Molecule 1; Cxcl1, C-X-C Motif Chemokine Ligand 1; Ccl2, C-C Motif Chemokine Ligand 2; NT5E, ecto-5′-nucleotidase; IFNy, Interferon gamma; MMP-3, Matrix metalloproteinase-3; MMP-9, Matrix metalloproteinase-9; COX-1, Cyclooxygenase-1; COX-2, Cyclooxygenase-2.

Two key immune cell populations—resident microglia and infiltrating monocyte-derived macrophages (MoDMs)—are critical mediators of the intracerebral immune response and shape the post-stroke environment. Microglia are a specialized, self-renewing macrophage population residing in the central nervous system (CNS), and are the first immune cells to respond to ischemic injury. In contrast, MoDMs are derived from circulating monocytes that migrate to the site of inflammation (9–13) **(** **Figure 1**). Once activated, microglia and MoDMs phagocytose debris, secrete cytokines, and present antigens to T cells, marking the induction of adaptive immunity. In this review, we discuss the unique roles of microglia and MoDMs in mediating the post-stroke response, and explore therapies targeting this response.

### Background: Microglia

Microglia and MoDMs are distinct cell populations that are involved in the innate immunological responses to brain injury and disease. Though microglia and MoDMs share similar phenotypes and functions, their ontology is unique, with microglia arising from erythromyeloid progenitors in the yolk sac and other mononuclear phagocytes, including dendritic cells, monocytes, and macrophages, arising from hematopoietic stem cells (14). During embryological development, microglia precursor cells migrate to the brain, where differentiation into microglia is driven by various external signals within the brain environment (15). Over the course of pre- to post-natal development, microglia display a variety of functions, including synaptic remodeling and maturation. In the adult brain, estimates of microglial abundance range from 5-20% (16). Though the full range of microglial programs is unknown, several key functions have been identified, including phagocytosis of myelin, modulation of neuronal activity, maintenance of oligodendrocyte progenitor cells, and immune defense (17, 18). Homeostatic microglia perform an extensive array of cellular processes which continually monitor the CNS for injury and pathogenic breach (19, 20). Upon detection of pathogenic breach or neuronal damage, microglia adopt an activated phenotype and phagocytose foreign invaders.

Microglia adopt distinct morphologies corresponding with their activation state. Bushy microglia have a larger soma surrounded by fewer and thicker cell processes when compared to homeostatic, ramified microglia. Amoeboid microglia are rounder with rare or even nonexistent cell processes. Upon transitioning from ramified to amoeboid morphology and migrating to the site of invasion/injury, microglia engage in the act of phagocytosis, one of their primary functions (15). Microglial migration is directed by molecular signals—including various cytokines and chemokines—which are released by injured neurons (21). Furthermore, microglia express key phagocytic receptors, including toll-like receptors (TLRs) and TREM-2, which recognize foreign pathogens and apoptotic cell debris (22, 23). The ultimate phagocytosis of these foreign materials and debris involves amoeboid microglia utilizing actin cytoskeleton reorganization to extend their processes around extracellular material, forming a phagosome (24, 25). The resultant phagosome then enters the endolysosomal pathway where the engulfed material is degraded. Microglia phagocytosis has been hypothesized to play a protective role in various disorders of the nervous system, including Alzheimer’s disease and Parkinson’s disease, by clearing pathological accumulations of amyloid beta and alpha-synuclein protein, respectively (26, 27). Additional important immune functions of microglia include the initiation of inflammatory cascades *via* cross talk with neurons, glial cells, and infiltrating monocytes. Microglia express MHC II and are capable of antigen presentation, although the extent to which they prime naïve lymphocytes vs participate in ongoing antigenic stimulation in the setting of stroke is unknown.

**Figure 2:**
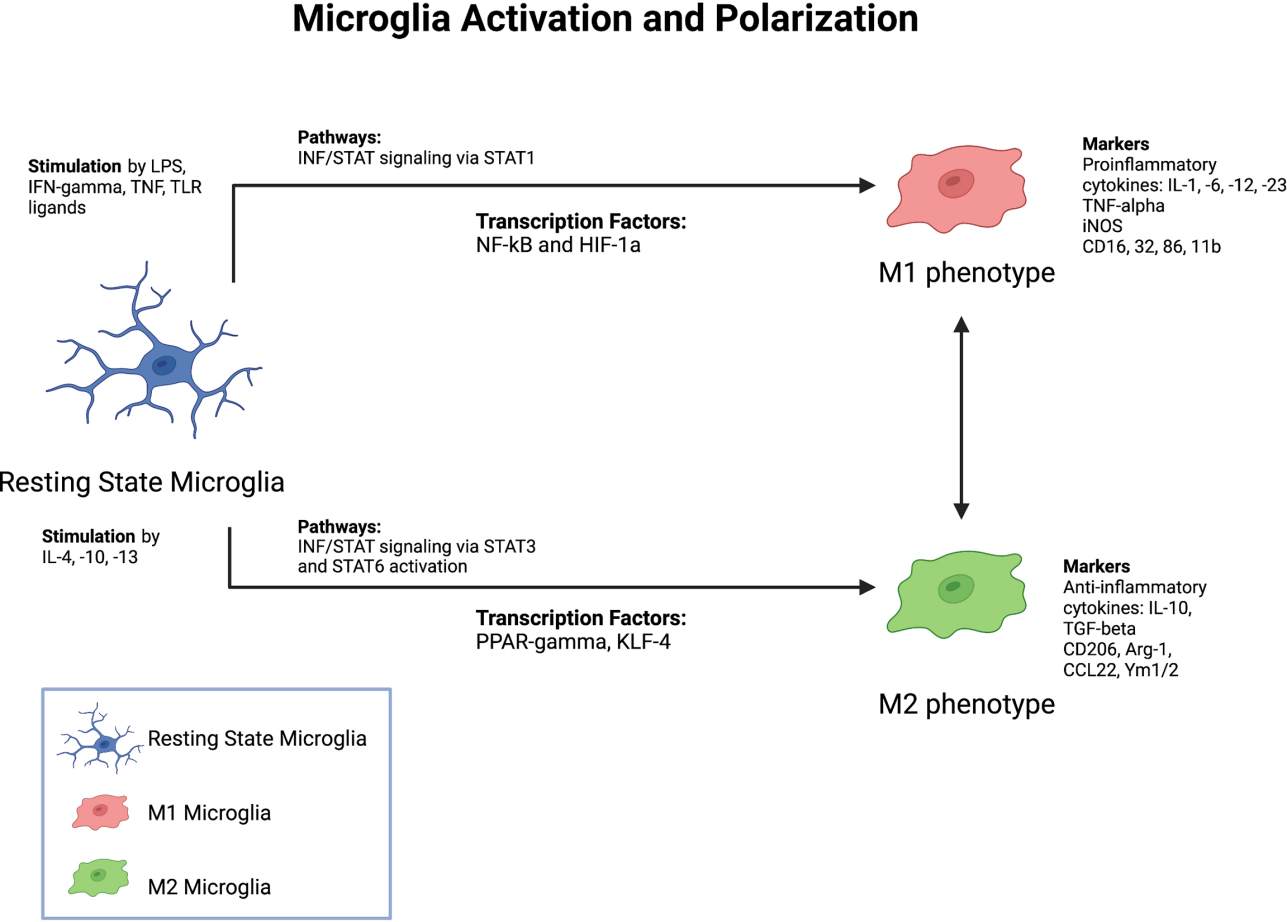
Factors Driving Microglia Activation and Polarization. Summary of major known stimulants, pathways, as well as markers of microglia activation and polarization.

Microglia have traditionally been classified as having a pro-inflammatory (M1) or anti-inflammatory (M2) phenotype (**Figure 2**). Microglia can also switch between the M1 and M2 phenotypes in response to changes in environmental conditions. Following ischemic stroke, oxidative stress triggers the activation of the antioxidative transcription factor nuclear erythroid related factor 2 (Nrf2) pathway (28). The Nrf2 pathway promotes microglia polarization to the anti-inflammatory M2 phenotype by increasing expression of anti-inflammatory genes such as NQO1 and HMOX1 (28). Enhancing Nrf2 pathway activity using various pharmacological compounds has been found to improve stroke outcomes in several preclinical studies (29, 30). Additionally, molecular compounds which suppress the NLRP3 inflammasome pathway have been found to promote phenotypic switching from the M1 to M2 activation state (31, 32). Furthermore, single-cell RNA sequencing has indicated that numerous activated microglia phenotypes exist based on clustering of transcriptomic data (33). Even within the M2 anti-inflammatory phenotype, several different activation subtypes such as M2a, M2b, M2c, and M2d, each with distinct functions in tissue repair and wound healing, have been identified (34). Clearly, activated microglia are a highly heterogenous cell population, with no clear consensus on how to define or differentiate various microglial subtypes. But while it is important to understand that M1 and M2 are not fixed phenotypes, the pro- and anti-inflammatory functions of these highly plastic phenotypes remain a useful framework for discussing the various roles of microglia in responding to ischemic stroke.

### Background: Monocyte-Derived Macrophages

Monocyte-derived macrophages (MoDMs) arise from monocytes, which, in turn, differentiate from hematopoietic stem cells in the adult bone marrow and are continuously regenerated throughout adulthood (35, 36). Although the specific role of MoDMs is dynamic, these cells generally function to produce proinflammatory factors, clear pathogens, and present antigens (37–40). On a cellular level, MoDMs can be distinguished from microglia based on gene transcript expression. Compared to MoDMs, microglia exhibit higher expression of CX3CR1, TREM2, and SIGLEC (41). Furthermore, several surface markers such as P2RY12, TMEM119, FCRLS, and intracellular markers such as SALL1 have been identified as specific to microglia (42, 43).

Whereas microglia remain confined to the CNS, monocytes are found in peripheral blood circulation, bone marrow, and the spleen. MoDMs and their precursors display classically activated (pro-inflammatory) or alternatively activated (anti-inflammatory) phenotypes, but are capable of intermediate activation states as well. Broadly, M1 MoDMs promote inflammation by releasing cytotoxic substances and inducing cell death whereas M2 MoDMs phagocytose cellular debris and release trophic factors that enhance recovery. As with M1 and M2 microglia subtypes, there is significant overlap in signature markers for M1 and M2 phenotypes amongst the MoDMs, suggesting there is a pro/anti-inflammatory continuum on which cells lie and shift dynamically. To further understand the MoDM phenotypes and their temporal expression post-stroke, it is important to further characterize the cells that will differentiate into these important players upon arrival to the ischemic site, the monocytes.

Human monocytes fall into three main subtypes based upon relative expression levels of the surface markers clusters of differentiation (CD) 14 and 16: classical monocytes (CD14^++^CD16^-^), intermediate monocytes (CD14^++^CD16^+^) and non-classical monocytes (CD14^+^CD16^++^) (44). CD14^++^CD16^-^ monocytes express the receptors CD64 and CD32, produce TNF-ɑ and IL-10, exhibit high peroxidase activity, and are primed for phagocytosis. CD14^++^CD16^+^ intermediate monocytes express the CCR5 receptor and have proinflammatory function, with comparable peroxidase activity to classical monocytes, but higher production of IL-1β, IL12, and TNFα. And finally, CD14^+^CD16^++^ monocytes have weak phagocytic activity and fail to produce TNF-ɑ or IL-1 (44–46). The classical monocytes (CD14^++^CD16^-^) are generally considered to be a short-lived pro-inflammatory subset involved in phagocytosis. Intermediate monocytes (CD14^++^CD16^+^) play a role in antigen presentation, apoptosis regulation, cytokine secretion and T-cell activation. Meanwhile, non-classical monocytes (CD14^+^CD16^++^) are involved in complement mediated phagocytosis and patrolling vascular endothelium for damage or infection (47, 48).

In mice, three major monocyte subtypes, which are homologous to the human subtypes, have been identified based on differential expression of the surface receptor Ly-6C: the classical Ly-6C^hi^CCR2^+^CD43^low^CX_3_CR1^low^monocytes, which represent approximately 2-5% of circulating white blood cells in healthy mice and are the first to arrive to inflamed tissues, the intermediate Ly-6C^hi^CD43^hi^ monocytes, and the non-classical, alternatively activated Ly-6C^low^CCR2^-^CD43^hi^CX_3_CR1^hi^ monocytes, which are longer-lived monocytes that surveil vasculature (49, 50). Ly6C^low^ mouse monocytes most closely correlate to CD14^+^CD16^++^ human monocytes and Ly6C^hi^ mouse monocytes are analogous to human CD14^++^ monocytes (44, 51, 52). While Ly6C^low^ cells have a half-life of nearly a week, Ly6C^hi^ cells have a half-life of less than one day (53).

Monocyte phenotypes are further distinguished by the relative presence of two chemokine receptors, C-X3-C Motif Chemokine Receptor 1(CX3CR1) and C-C Motif Chemokine Receptor 2 (CCR2), which are found on both murine and human cells (50, 54–57). Both human and mouse classical monocytes express high levels of CCR2 and low levels of CX3CR1 while non-classical monocytes of both species express high CX3CR1 and low levels of CCR2. The relative presence of these two key receptors is the basis of the migration and homing mechanism of monocytes into areas of inflammation.

Monocyte migration is directed by molecular signals, including cytokines and chemokines, which are released by injured neurons as well as astrocytes and microglia (21, 58) (**Figure 1**). Within peripheral blood circulation, CC-chemokine ligand 2 (CCL2) and ligand 7 (CCL7) are the key chemokines that bind to CCR2 and facilitate Ly6C^hi^ classical monocyte recruitment (59). The recruitment of Ly6C^low^ monocytes is dependent upon the CX_3_C-chemokine ligand 1 (CX_3_CL1), which is expressed in tissues as well as the marginal zone of the spleen. Other chemokine monocyte receptors, including CCR1, CCR5, CCR6, CCR7, CCR8 and CXCR2 have also been reported to be involved in monocyte recruitment, though their roles are less prominent (60–66).

Once activated, classical monocytes travel through the bloodstream to the site of inflammation, where they adhere to the endothelial surface through the binding of integrins and other adhesion molecules, and extravasate across the blood vessel wall into the area of inflamed tissue. Key adhesion molecules that have been described include, L-selectin, P-selectin glycoprotein ligand 1 (PSGL1), platelet endothelial cell adhesion molecule (PECAM1), macrophage receptor 1(MAC1), lymphocyte function-associated antigen 1 (LFA1), and very late antigen 4 (VLA4) (67–70). It has been reported that signaling in classically activated monocytes through the CCR2-CCL2 axis alters the conformation of VLA-4, leading to higher affinity interaction with its receptor vascular cell adhesion molecule-1 (VCAM-1) and ultimately monocyte transmigration into the infarcted tissue (71). On the other hand, alternatively activated monocytes bind to the endothelium *via* CX3CR1-CCL3 and transmigrate in an LFA1/Intercellular Adhesion molecule-1 (ICAM1) dependent manner (57).

Upon arrival to the site of inflammation, the undifferentiated classical monocytes begin to exhibit changes in immune phenotype by downregulating Ly6C and upregulating F4/80, characteristic of mature phagocytes (72). They also progressively acquire expression of alternatively activated macrophage markers, such as YM-1 and arginase-1 (12). Further differentiation results in upregulation of pro- or anti-inflammatory characteristics depending on key molecular pathways that affect gene expression and cellular metabolism (73). These include the P13K/AKT, PPARs, MYC, NOTCH, and IRFs pathways (43).

The classically activated M1 MoDMs contribute to tissue degradation and T cell activation (through expression of MHC-II) and are distinguished by secretion of proinflammatory cytokines TNF-alpha and IL6. The alternatively activated M2 MoDMs express YM-1 and arginase-1, secrete anti-inflammatory cytokines such as IL-10, and are involved in wound healing, angiogenesis, and tissue fibrosis (71). M1 MoDMs display a higher expression of markers CD38, CD274, CD197, CD54, CD82, CD86, and Slamf7, while M2 MoDMs express higher amounts of CD163, CD206, and Neurophilin (74). Cellular metabolism of these two subtypes also differs, with M1 macrophages relying upon aerobic glycolysis whereas M2 macrophages depend on the TCA cycle and oxidative phosphorylation (43). Through manipulation of enzymes involved in these pathways, such as Pyruvate Dehydrogenase (PDH), the M1/M2 phenotype dynamic could be shifted.

Conventionally, there has been a linear understanding in monocyte-to-macrophage differentiation, where the classical or pro-inflammatory monocytes only differentiate into M1 macrophages and the alternatively activated or anti-inflammatory monocytes only differentiate into M2 macrophages. This understanding of monocytes possessing pre-determined differentiation states once arriving at the site of inflammation is now in question, as studies have shown that in the absence of anti-inflammatory monocytes from the bone marrow, M2 macrophages have been identified in the infarcted brain (75). These findings highlight the need for further research into the signals and timing involved in monocyte-to-macrophage differentiation.

## Recruitment and Activation of Microglia and MoDMs During Ischemic Stroke

At the onset of ischemic stroke, activated, amoeboid microglia rapidly migrate to the site of ischemic injury. The reduction in cerebral blood flow to the area of injury initiates a sequence of events often referred to as the ischemic pathway, which is notable for energy depletion and glutamate excitotoxicity leading to cell death (76). The ischemic core, defined as the region in which irreversible cell death has occurred, is characterized by elevated ion concentrations and glutamate levels as well as tissue acidosis (76). Elevated glutamate levels drive the release of microglial chemoattractants, such as ATP, CCL21, CXCL10, and IL-1*β*, leading to microglial recruitment (77). Imaging studies of ischemic stroke mouse models have found that within the first 24 hours of ischemia, and as early as thirty minutes after stroke onset (78) activated microglia (as characterized by an amoeboid morphology) populate the area of injury (19, 79). Adenosine triphosphate (ATP) release by dying neurons serves as a chemoattractant which activates purinergic receptors, such as P2Y12, on microglia and guides their directional migration to the site of injury (80). Downstream signaling pathways then enable chemotaxis *via* adhesion disassembly (81). Once microglia reach the site of injury, they are activated by damage-associated molecular patterns (DAMPs). High mobility group box 1 (HMGB1) is one such DAMP secreted by dying neurons following ischemic stroke. HMGB1 binds to TLR4 on microglia and induces production of pro-inflammatory cytokines, including interleukin (IL)-6 and tumor necrosis factor-alpha (TNF-alpha). Heat shock proteins, such as Hsp70, also activate microglia *via* nuclear factor-kappa B (NF-kB) activity (21). Microglia in turn secrete matrix metalloproteinase (MMP)-9, inducible nitric oxide synthase (iNOS), and reactive oxygen species (ROS) (81). These activities are temporally coordinated as some studies suggest that during the earlier stages of injury, microglia primarily promote tissue repair and reconstruction of the extracellular matrix (82, 83). In the later stages of injury, microglia shift towards a pro-inflammatory phenotype in which they release inflammatory cytokines and generate ROS (82, 83). This transition is not absolute, however, as other authors have shown that activated microglia can also secrete anti-inflammatory cytokines, such as transforming growth factor-beta (TGF-β) and IL-10 (84) at later timepoints (**Figure 2**).

In a transient ischemic rat model, microglia activation was evident 3.5 hours after the onset of ischemia (79). In a permanent ischemic mouse model, microglia featuring hypertrophic cell bodies and shortened processes were observed as early as the 30-minute time point (79, 85). Activated microglia are also recruited to the penumbra, the potentially salvageable region of brain tissue surrounding the ischemic core. In the penumbra, activated microglia are notably associated with blood vessels. Some studies have suggested that perivascular activated microglia play an important role in blood vessel repair as well as promoting the integrity of the BBB, which is compromised during ischemic stroke (86, 87). However, the role of perivascular activated microglia in mitigating tissue injury remains controversial, with other studies suggesting that they promote blood vessel disintegration (88).

Gender and aging also influence the microglial response to stroke. Mouse model studies have demonstrated that resting state male microglia exhibit higher expression of inflammatory genes regulated by the NF-kb transcription factor compared to female microglia, and male mice subjected to cerebral ischemia developed larger infarcts than female mice (89, 90) The difference in infarct size may be due to increased expression of genes significant for cellular plasticity in female microglia compared to male microglia (91). Additionally, aged mice have been found to experience more severe neurologic deficits following MCAO as well as increased serum levels of inflammatory cytokines compared to young mice (92). One hypothesis for this finding is the upregulation of IRF5 signaling which occurs with aging (92). Microglia in aged mice have been found to resist phenotypic transformation to an M2 anti-inflammatory state in response to IL-4 (93). Therefore, male sex and increased age may both impair anti-inflammatory aspects of the microglia response to ischemic injury.

Mobilization of monocytes from the bone marrow to the blood and ultimately into the infarcted tissue is dependent on the CCL2/CCR2 axis (59, 94, 95). CCL2 is produced by astrocytes and microglia in states of hypoxia, as it is under direct transcriptional control of Hypoxia-inducible factor 1-alpha (HIF-1alpha) (58). Because CCL2 exclusively binds to CCR2, which is only expressed on the classically activated, pro-inflammatory monocyte subset, this is the subset recruited to the ischemic site, a finding supported in experimental models of brain ischemia and hemorrhage (10, 72, 96). Garcia-Bonilla et al. utilized ischemic stroke models with CX3CR1^GFP/+^CCR2^RFP/+^ bone marrow (BM) chimeric mice to study the effects of CCR2 and CX3CR1 on monocyte/macrophage recruitment following stroke and showed that non-classical, alternatively activated CX3CR1^+^ monocytes/macrophages were absent in the brain of CCR2 null mice. Furthermore, they found that while circulating hematogenously-derived alternatively-activated monocytes were absent in NR4A1-deficient mice (a nuclear receptor responsible for the differentiation and survival of non-classical, alternatively activated monocytes), these mice still had increased alternatively-activated monocytes in the brain 14 days after stroke occurred (51, 75). This led the authors to conclude that the alternatively-activated monocytes were, in fact, derived from the classically-activated monocytes once they had arrived at the site of ischemia, and were not produced *de novo* in the bone marrow.

Breakdown of the BBB facilitates the transmigration of cells including monocytes from the periphery into the site of injury. Nadareishvili et al. quantitatively measured gadolinium leakage through serial MRIs to assess the degree of BBB disruption after thrombolytic therapy for acute stroke and found that increased BBB permeability was associated with worse outcomes in patients independent of the severity and size of stroke. In fact, for every 1% increase in BBB permeability there was a 75% decrease in the chance of a good long-term functional outcome (97). BBB permeability has been shown to occur in two phases: reversible disruption occurs early after ischemic onset and is driven by the release of MMP2, later BBB disruption is mediated by MMP-3, MMP-9, and cyclooxygenases days after the ischemic insult (98). This second wave of BBB disruption allows for the infiltration of systemic immune cells, including neutrophils, dendritic cells, T-cells, NK cells and monocytes. Endothelial activation also results in chemokine release and upregulation of adhesion molecules, both of which facilitate recruitment and transmigration of circulating monocytes. Endothelial and tissue resident macrophages regulate the infiltration of monocytes and neutrophils through the production of cytokines such as granulocyte macrophage-colony stimulating factor (GM-CSF) (99). Ischemia also triggers expression of adhesion molecules such as selectins, integrins, and intercellular and vascular adhesion molecules along the microvasculature, which facilities rolling and high affinity interactions for firm adhesion of circulating leukocytes (100).

Anatomically, the choroid plexus may be a particularly important route of monocyte recruitment and ingress into the infarcted brain region. Through gene expression studies and the use of chimeric mouse models, Ge et al. found that the choroid plexus responded to stroke by upregulation of several key mediators of MoDM trafficking (such as Vcam1, Madcam1, Cxcl1, CCL2, NT5E, and IFNy) which resulted in increased trafficking of MoDMs into the choroid plexus and CSF. If primed for the M2-phenotype *in vitro* using treatment with macrophage-colony stimulating factor (M-CSF), IL4, and IL13 prior to administration into the lateral ventricle ipsilateral to the ischemic lesion, MoDMs homed to the area of ischemia and promoted post-stroke recovery and improved cognition (101).

Once monocytes have infiltrated the area of ischemia, the ischemic environment promotes their differentiation into macrophages. While CCR2^+^ monocytes retain a round, amoeboid phenotype and are limited to the ischemic core, CX3CR1^+^ monocyte/macrophages can adopt three different phenotypes in the ischemic brain based on where they are localized relative to the infarct core: in the penumbra, they generally take on a ramified phenotype similar to homeostatic microglia, while in the infarct region, they take on an amoeboid, phagocytic macrophage phenotype, and when associated with blood vessels, resemble perivascular macrophages (75). Using the C-X-C Chemokine Receptor Type 4 (CXCR4) signature to trace cells of hematopoietic stem cell origin, it was found that monocyte infiltration occurs in both the peri-infarct and infarct areas after transient MCAO (102). In a photothrombosis infarct model, infiltration primarily occurred in the peri-infarct region. Interestingly, in CXCR4 knockout mice that underwent photothrombosis, monocyte infiltration and microglial proliferation were both reduced, suggesting that MoDMs are responsible for microglial repopulation of the infarct core. Furthermore, the authors also demonstrated that MoDMs were the main source of microglia-activating mediators following photothrombosis and maintained microglial activation in the peri-infarct region until they were cleared (102).

## Activity and Function of Microglia and MoDMs During the Three Stages of Ischemic Stroke

**Figure 3:**
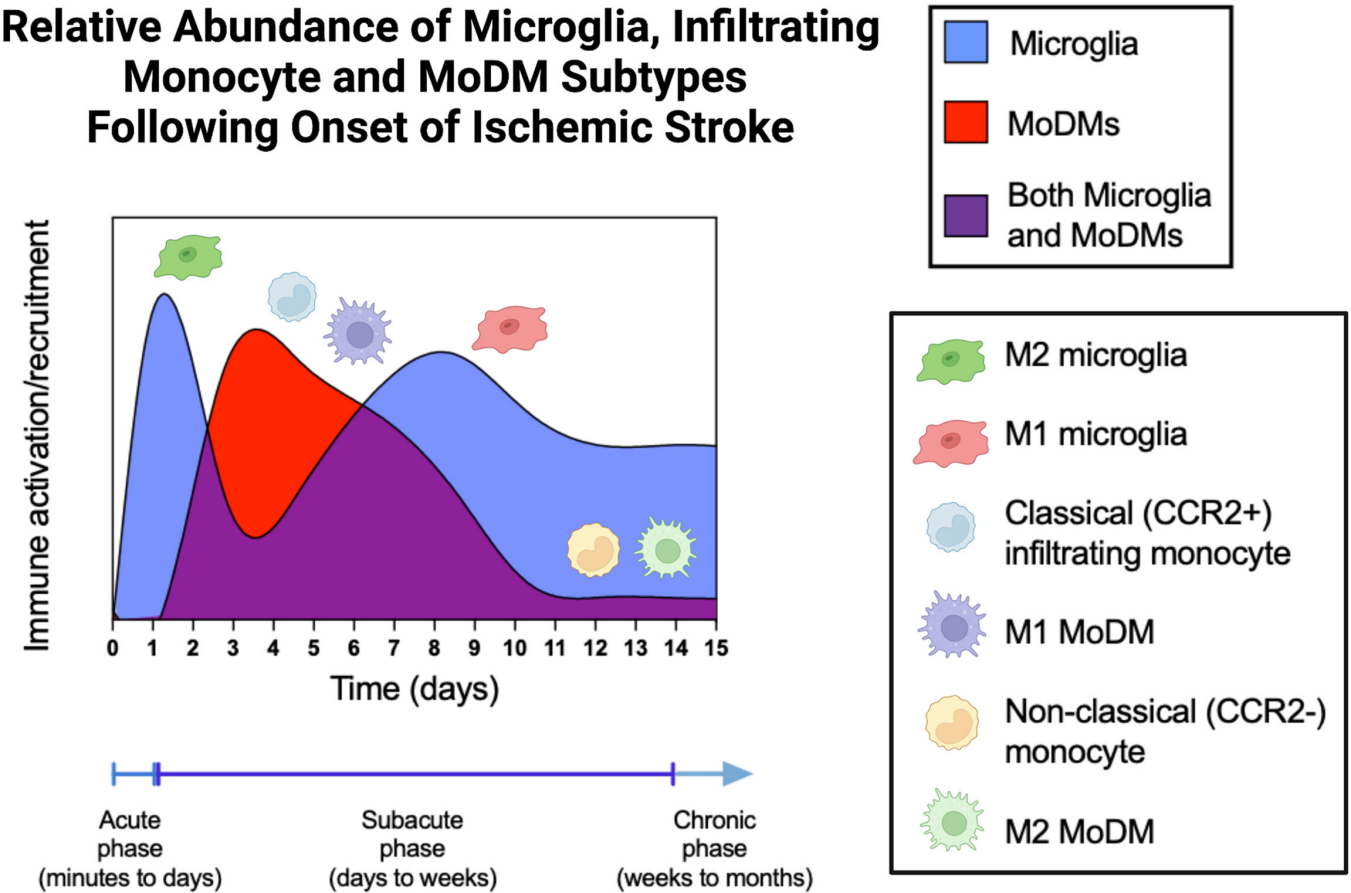
Relative Abundance of Microglia, Infiltrating Monocyte, and MoDM Subtypes Following Onset of Ischemic Stroke. During the acute phase of stroke, microglia are activated and predominantly found in the M2, anti-inflammatory phenotypic state. These M2 microglia then wane during the early subacute phase, giving rise to the M1 microglia during the late subacute and chronic stages. Classical monocytes (CCR2^+^, Ly6C^high^ cells in mice, CD14^++^ CD16^-^ cells in humans) infiltrate the brain parenchyma from days 3-5 and differentiate into M1 pro-inflammatory MoDMs. After day 7, the quantity of MoDMs slowly returns to baseline levels, which are reached by day 14. During this time, non-classical monocytes (CCR2^-^, Ly6C^low^ cells in mice, CD14^+^ CD16^++^ cells in humans) and the differentiated M2 MoDMs predominate. MoDM, monocyte derived macrophage; CCR2, C-C Motif Chemokine Receptor 2.

Stroke is clinically staged into the acute, subacute, and chronic periods (**Figure 3**). The acute period is generally defined as the period of minutes to days following the ischemic insult, while the subacute period refers to the time from days to weeks following stroke, and the chronic period refers to the time period from weeks to months and beyond. The chronic stage can last for years and continue for the remainder of a patient’s life (8). Depending on the stage of stroke, microglia and MoDMs are preferentially polarized towards different activation states in order to carry out specialized functions. Mismatch between these activation states and the timing of recovery, or inflammation that becomes chronic can both be detrimental to long-term outcomes.

### The Acute Phase

During the first 24 hours after ischemic injury, activated microglia predominantly exist in an anti-inflammatory state, as indicated by increased expression of CD206 and Ym1 (103). Additionally, canonical M2 markers, including CD206, Arg1, CCL22, Ym1/2, IL-10, and TGF-β are highly expressed starting 1-3 days after MCAO (104). M2 marker expression peaks around 3-5 days post-injury, and begins to decrease at seven days, finally returning to pre-ischemic insult levels by the subacute phase of stroke. M2 polarization is influenced by the activation of the transcription factor, peroxisome proliferator-activated receptor γ (PPARγ), as well as stimulation by IL-4 and IL-13 cytokines (105, 106). Activated M2 microglia secrete anti-inflammatory cytokines, such as IL-10, TGF-β, IL-4, IL-13, and insulin-like growth factor 1 (107–109). M2 microglia demonstrate increased phagocytic activity and are speculated to clear cell debris and injured tissue from the infarct area. These cells also promote tissue repair and recovery by promoting neurogenesis *via* nerve growth factor production, promoting angiogenesis *via* IL-8 and vascular endothelial growth factor (VEGF) production, and enhancing axonal regeneration *via* VEGF/TGF-*β*/IGF-1 production (110). The protective effect of M2 microglia during the acute phase of stroke has been indirectly demonstrated by the fact that microglia depletion following ischemic stroke exacerbates neuronal injury (111).

In contrast, the pro-inflammatory M1 phenotype is rarely observed in the ischemic core during the first 24 hours after stroke. In murine models of ischemic stroke, low expression of M1 markers iNOS, CD16, CD32, CD86, and CD11b has been observed in the three-day period following stroke onset (104). At these early timepoints, M1 or amoeboid microglia are concentrated in the penumbra. M1 microglia first appear in the ischemic core 24 hours after infarction, and their numbers peak at 14 days (108). The differentiation of microglia into the M1 phenotype following ischemic injury is prompted through activation of the NF‐κB transcription factor (112). NF-kB prompts the secretion of proinflammatory cytokines, such as IL-1β, IL-6 and TNF-α, as well as the production of inducible nitric oxide synthase (iNOS) and reactive oxygen species (ROS) (109). These combined processes result in secondary brain damage by exacerbating neuronal death and neuroinflammation, causing death of oligodendrocytes and oligodendrocyte progenitor cells, suppressing remyelination, and inhibiting neural precursor cell proliferation (113).

In the immediate peri-infarct region, microglia adopt a bushy morphology and demonstrate lower phagocytic activity (110, 113). The exact functions of microglia bearing different morphologies, including whether they exacerbate neuronal injury or promote neuroinflammation, is an area of active research. Additionally, it is important to note that microglia activation is not limited to the infarct core. Activated microglia are also present in the penumbra as well as remote brain regions which are functionally or anatomically connected to the primary injury site (22, 113, 114). This shifting spatial pattern of M1 phenotype from penumbra to core may play a role in limiting initial damage as some studies suggest that M1 microglia constrain expansion of the core (115). The M1 phenotype then predominates in the subacute phase.

MoDMs are sparse in the CNS during the acute phase of ischemic stroke. However in mice, the number of classically activated (Ly6C^hi^) monocytes has been shown to be increased in the peripheral blood circulation during the acute phase (within 3 hours) and decreased to pre-ischemic levels in the sub-acute and chronic phases (from 1 to 7 days following ischemic onset) (96). Similarly, clinical studies have shown that there is an increased number of intermediate and classical monocytes in the blood circulation at acute and sub-acute phases of stroke, with decreased non-classical monocytes in blood circulation at these stages (116). In those patients with progressive infarction and severe injury, increased numbers of intermediate monocytes coupled with decreased numbers of non-classical monocytes were identified in the peripheral blood (117).

### The Subacute Phase

At approximately one week following ischemic stroke, the number of M1 microglia begins to dramatically increase with a concomitant decrease in M2 microglia (108). This transition is referred to as an M2-to-M1 phenotypic shift, and M1 microglia remain the predominant activated form during the chronic phase of stroke. The continued release of proinflammatory factors from M1 microglia has been hypothesized to contribute to neuronal death, and persistent activation during the chronic phase of stroke may impair long term recovery (83, 118–120).

Like microglia, MoDMs also adopt time frame-dependent activation states that can amplify or attenuate the inflammatory response. Because these activated monocytes must exit the bone marrow, travel through the bloodstream, and cross the blood-brain barrier before differentiating into effector macrophage subsets, the presence of MoDMs peaks days after the inciting event (72, 121). In fact, studies using fluorescent cell tracking and magnetic resonance imaging to track MoDMs have found that they do not significantly contribute to the inflammatory response in the infarcted tissue until 3-7 days after ictus (9, 122, 123). In a murine study performed by Schilling et al. using GFP-stained hematogenous macrophages, GFP+ cells were present no earlier than the fourth day post-stroke (124, 125). This finding was supported in other transient and permanent MCAO animal models using MR imaging and iron-oxide particles (USPIO) to track MoDMs, which showed increased signals in the ischemic zone for 7 days following stroke, with the peak signals being noted on day 4 (126).

The majority of monocytes initially recruited to the brain after stroke are Ly-6Chi (CCR2+) cells, which differentiate primarily into M1 tissue macrophages in the stroked hemisphere and promote inflammation. Classically activated monocytes appear to be the dominant phenotype found in the infarct core during the subacute phase (3-5 days after stroke) (75). As they persist in the site of inflammation, however, these cells lose their Ly-6C and CCR2 expression and begin to release VEGF and TGF-β, facilitating angiogenesis and neuroprotection (127). Studies have termed this effect the “dead cell clearance” hypothesis, which posits that upon exposure to apoptotic cells, classically activated M1-like macrophages switch toward the alternatively activated M2 phenotype (128). Thus, by day 7, anti-inflammatory monocytes and MoDMs define the post-stroke setting (117). The presence of infiltrating monocytes in the subacute phase has been associated with decreased risk of hemorrhagic transformation (72). Furthermore, MoDMs have been shown to have a beneficial role in post-stroke recovery through modulating detrimental acute and long-term microglial-mediated inflammation (129).

### The Chronic Phase

The chronic phase of stroke refers to the period of weeks to months after onset of the initial ischemic event. During this phase, microglia activation persists in the pro-inflammatory M1 activation state, and is associated with pathologic inflammation, neurodegeneration, and decreased neuroplasticity. The biological mechanisms underpinning the persistence of M1 microglia are not fully understood, but some known implicated pathways include increased activity of transcription factors such as Irf5 as well as downregulation of the CREB-C/EBPβ cascade (104). The pro-inflammatory cytokines released by M1 microglia damage nearby neurons, prompting the release of DAMPs which further perpetuates the inflammatory response. The long-term consequences of prolonged inflammation include dysfunctional or diminished tissue repair, synaptic plasticity, neurogenesis, axonal and dendritic spine regeneration, neural network reorganization, interhemispheric connections, and neuroplasticity (118, 130) In a preclinical study of MCAO mice, peripheral administration of IL-13 was found to induce an anti-inflammatory microglial response, resulting in improved gait and sensorimotor deficits at seven and 14 days post-stroke, respectively (130). Therefore, while interventions in the acute phase of stroke may be important for limiting initial ischemic damage, interventions in the chronic phase could potentially improve long-term neurofunctional outcomes.

On the contrary, during the late subacute to chronic phase (14-28 days post-stroke), alternatively activated monocytes predominate and primarily differentiate into the M2 anti-inflammatory MoDM phenotype (75) (**Figure 3**). Consistent with this finding, MoDMs have been shown to contribute to long-term spontaneous functional recovery (131, 132). MoDMs play a critical role in clearing debris and dead cells (99). Using an anti-CCR2 antibody, MC-21, Watannanit et al. were able to block monocyte recruitment and found that this resulted in decreased tissue expression of the anti-inflammatory genes TGFβ, CD163, and Ym1 and functional inability of mice to recover long-term (13). Yet, there are conflicting data in other models (133–135). In an intracerebral hemorrhage model, Hammond et al. reported that classical monocytes exacerbated acute disability (135). Using clodronate liposomes to deplete peripheral macrophages, Ma et al. found that under conditions of macrophage depletion, there was decreased demyelination and brain atrophy in the ipsilateral striatum and enhanced focal microvessel density in the peri-infarct region, all of which have been correlated with longer survival times in ischemic stroke patients (134, 136). Further long-term studies of the effects of MoDMs on recovery are needed to better understand these discordant findings regarding the activity and function of MoDMs in the chronic stage. These conflicting findings further highlight the limitations of M1 and M2 classification and future studies will be needed to move beyond phenotypic descriptions and better understand specific activities and pathways that promote recovery or injury at each stage.

## Microglia and MoDMs Directed Treatments for Ischemic Stroke

Clinical therapies for stroke are currently focused on salvaging the penumbra in the acute phase *via* a combination of reperfusion techniques including intravenous thrombolytics and mechanical thrombectomy. Treatment strategies for the ensuing brain edema are mainly supportive, involving interventions such as hyperosmolar therapy, corticosteroids, hyperventilation and CSF diversion to reduce intracranial pressure during the acute swelling period. By the subacute and chronic stages, the treatment focus shifts to recovering function through physical and neuropsychological rehabilitation (137). At present there are no approved therapies that target the inflammatory pathways to limit secondary injury. Several pharmacological agents have been explored for their ability to promote neuroplasticity and neurogenesis during the later stages of stroke, including antidepressants, amphetamines, and neurotrophins (138). However, randomized controlled trials for these agents have failed to meet their efficacy endpoints (139).

The induction or promotion of the anti-inflammatory M2 microglia phenotype is one treatment strategy that has been employed in both preclinical and clinical studies to decrease inflammation in the acute and subacute phases and promote brain plasticity through in the chronic phase (113). Promotion of the M2 phenotype can be accomplished by administering molecular compounds which activate specific cell signaling pathways, such as the STAT3, AMPK, and PPARγ pathways (140–142). In preclinical studies, intravenous injection of compounds, such as xuesaitong (a Chinese patent medicine) and Ac2-26 (an annexin/lipocortin 1-mimetic peptide), as well as intraperitoneal injection of compounds such as recombinant human fibroblast growth factor 21 (rhFGF21 and melatonin, have been reported to promote the M2 microglia phenotype by modulating cell signaling (143–146) (**Table 1**). HP-1c, an activator of the AMPK and Nrf2 pathways, as well as CDDO-EA, an activator of the Nrf2 pathway, also promote the M2 microglia phenotype (29, 30). Inhibition of polarization to the M1 phenotype *via* inhibition of the PDE5 pathway is another treatment which has shown preclinical success. Administration of sildenafil, a PDE5 inhibitor, after MCAO has been found to decrease the number of M1 microglia during the later stages of stroke as well as reduce the extent of the ischemic lesion (149). Nitric oxide (NO) and hydrogen sulfide (H2S)-releasing hybrid) (NOSH-NBP) has been similarly found to promote the M2 microglia phenotype in murine models of cerebral ischemia (152). Furthermore, clinical trials evaluating the safety and efficacy of NBP in mild to moderate ischemic stroke patients are ongoing with results still pending (**Table 2**). Minocycline is an antibiotic found to promote the M2 microglia activation state in preclinical studies and has demonstrated some preliminary success for improving neurofunctional recovery following acute ischemic stroke in early clinical trials (161, 162). The MINOS (minocycline to improve neurological outcome in stroke) study was a phase 1 open-label, dose-finding study which found that minocycline could be safely tolerated in acute stroke patients at intravenous doses of up to 10 mg/kg (163). In a multicenter prospective randomized open-label pilot study of intravenous minocycline in a small sample of acute stroke patients, Kohler et al. reported that minocycline was safe but not efficacious. However, this study was not powered to reliably identify modest differences in clinical outcomes (164). In a small, open-label evaluator-blinded trial, Amiri-Nikpour et al. reported a gender-dependent effect on minocycline neuroprotection in ischemic stroke, noting improved clinical outcomes (lower National Institutes of Health Stroke Scale (NIHSS) scores) in minocycline-treated male patients, but no significant difference in minocycline-treated female patients (165). Of note, in these clinical trials, minocycline was administered as an oral or intravenous formulation to both ischemic and hemorrhagic acute stroke patients exclusively during the acute phase (161, 164–166). Additional clinical trials which test the efficacy of such pharmacological compounds are needed to determine whether modulating microglia phenotype can lead to improvement in neurologic outcome post ischemic stroke.

**Table 1:** Pre-clinical Studies Investigating Microglia, Monocyte, and MoDM Phenotype Modulation.

Author Year	Study Title	Model	Treatment	Results
Jin 2014 (147)	Improvement of functional recovery by chronic metformin treatment is associated with enhanced alternative activation of microglia/macrophages and increased angiogenesis and neurogenesis following experimental stroke	male CD-1 mice given tMCAO	IP metformin given daily at 50 mg/kg	Metformin treatment improved neurofunctional recovery, promoted microglia polarization to the M2 phenotype, enhanced angiogenesis, and enhanced neurogenesis
Tang 2014 (148)	CX3CR1 deficiency suppresses activation and neurotoxicity of microglia/macrophage in experimental ischemic stroke	CX3CR1⁻/⁻ C57BL/6 mice with tMCAO	CX3CR1 KO mice exposed to 90 min transient focal ischemica	CX3CR1 KO reduced infarct volume, attenuated neurological deficits, reduced proliferation of macrophages/microglia in ipsilateral hemisphere, reduced ROS generation, and reduced microglia/macrophage inflammatory response
Moretti 2016 (149)	Sildenafil, a cyclic GMP phosphodiesterase inhibitor, induces microglial modulation after focal ischemia in the neonatal mouse brain	C57BL/6 mice with permanent MCAO	IP sildenafil citrate given 5 min after MCAO	Sildenafil treatment reduced lesion size and promoted microglia polarization to the M2 phenotype
Shu 2016 (150)	Ginkgolide B Protects Against Ischemic Stroke *Via* Modulating Microglia Polarization in Mice	male C57BL/6J mice with tMCAO	IP ginkgolide B given twice daily after reperfusion (1.75 mg/kg, 3.5 mg/kg, and 7.0 mg/kg)	Gingkgolide B treatment promoted microglia polarization to the M2 phenotype, reduced infarct volume, and attenuated neurological deficits
He 2017 (151)	Thiamet G mediates neuroprotection in experimental stroke by modulating microglia/macrophage polarization and inhibiting NF-κB p65 signaling	male C57BL/6 mice with tMCAO	IP thiamet G given at 20 mg/kg each day for 3 days before tMCAO	Thiamet G treatment reduced infarct volume, attenuated neurological deficits, suppressed microglia/macrophage activation, and promoted microglia polarization to M2 phenotype
Ji 2017 (152)	NOSH-NBP, a Novel Nitric Oxide and Hydrogen Sulfide- Releasing Hybrid, Attenuates Ischemic Stroke-Induced Neuroinflammatory Injury by Modulating Microglia Polarization	C57BL/6 mice with tMCAO	PO drugs (NO-NBP, H2S-NBP, PTIO + NOSH-NBP, BSS + NOS-NBP, NOSH-NBP) given directly after reperfusion and once daily	NO-NBP, H2S-NBP, and NOSH-NBP treatments attenuated neurological dysfunction, decreased infarct volume, and decreased neuronal apoptosis; NOSH-NBP treatment was more effective than NO-NBP and H2S-NBP treatments; carboxy-PTIO (NO scavenger) and bismuth (III) subsalicylate (H2S scavenger) decreased the beneficial effects of NOSH-NBP
Qin 2017 (140)	Fingolimod Protects Against Ischemic White Matter Damage by Modulating Microglia Toward M2 Polarization *via* STAT3 Pathway	C57BL/6J mice with bilateral carotid artery stenosis	IP FTY720 given for 3, 10, or 30 consecutive days	FTY720 treatment ameliorated disruption of white matter integrity, attenuated microglia-mediated neuroinflammation, increased oligodendrocytogenesis, promoted microglia polarization to the M2 phenotype, and reduced cognitive decline
Schmidt 2017 (153)	Targeting Different Monocyte/Macrophage Subsets Has No Impact on Outcome in Experimental Stroke	C57BL/6 mice with tMCAO	IP clodronate liposomes daily, IV M1- or M2-macrophages transplanted after reperfusion	No effect on neurological outcomes observed
Jiang 2018 (154)	Exosomes from MiR-30d-5p-ADSCs Reverse Acute Ischemic Stroke-Induced, Autophagy-Mediated Brain Injury by Promoting M2 Microglial/Macrophage Polarization	male Sprague-Dawley rats with permanent MCAO	IV exosomes from miR-30d-5p-overexpressing ADSCs given at 80 ug per 2 mL after MCAO	Exosome treatment inhibited microglia polarization to the M1 phenotype and reduced infarct volume
Wang 2018 (29)	A Dual AMPK/Nrf2 Activator Reduces Brain Inflammation After Stroke by Enhancing Microglia M2 Polarization	Sprague-Dawley rats given tMCAO or pMCAO	IV HP-1c given at 1 mg/kg after MCAO	HP-1c promoted microglia polarization to the M2 phenotype, reduced infarct volume, improved neurological deficits, and reduced macrophage/microglia accumulation in ipsilateral hemisphere
Gelosa 2019 (155)	Improvement of fiber connectivity and functional recovery after stroke by montelukast, an available and safe anti-asthmatic drug	male CD1 mice with permanent MCAO	IP montelukast sodium powder administered 3 days before MCAO	Montelukast treatment reduced ischemic lesion volume, enhanced oligodendrocyte progenitor cell proliferation, and promoted microglia polarization to the M2 phenotype
Kolosowska 2019 (130)	Peripheral Administration of IL-13 Induces Anti-inflammatory Microglial/Macrophage Responses and Provides Neuroprotection in Ischemic Stroke	male BALB/cOlaHsd mice with permanent MCAO	IV IL-13 (1, 2, or 5 μg/animal) given following recovery from anesthesia	IL-13 treatment decreased ischemic lesion volume, reduced leukocyte infiltration, and promoted microglia polarization to the M2 phenotype
Li 2019 (143)	Xuesaitong May Protect Against Ischemic Stroke by Modulating Microglial Phenotypes and Inhibiting Neuronal Cell Apoptosis *via* the STAT3 Signaling Pathway	C57BL/6 mice with tMCAO	IV xuesaitong given directly after reperfusion for 14 consecutive days	Xuesaitong treatment reduced infarct volume, improved neurological outcome, promoted microglia polarization to the M2 phenotype, reduced the secretion of pro-inflammatory cytokine IL-1β, and increased secretion of trophic factors IL-10 and TGF-β1
Song 2019 (156)	M2 microglia-derived exosomes protect the mouse brain from ischemia-reperfusion injury *via* exosomal miR-124	male ICR mice with tMCAO	IV M2-derived exosome after reperfusion, 100 ug/day for 3 days	M2-derived exosome treatment attenuated neuronal apoptosis, reduced infarct volume, and attenuated neurological deficits
Yang 2019 (157)	Remote Postischemic Conditioning Promotes Stroke Recovery by Shifting Circulating Monocytes to CCR2+ Proinflammatory Subset	C57BL/6 mice with tMCAO	splenocytes collected from CCR2 KO mice transferred *via* retro-orbital injection to asplenic C57BL/6 mice; mice then subjected to tMCAO followed by remote limb conditioning (RLC) 2 hours post- reperfusion	RLC promoted pro-inflammatory subsets of monocytes, reduced infarct size, and improved functional recovery
Ye 2019 (158)	Meisoindigo Protects Against Focal Cerebral Ischemia-Reperfusion Injury by Inhibiting NLRP3 Inflammasome Activation and Regulating Microglia/Macrophage Polarization *via* TLR4/NF-κB Signaling Pathway	C57BL/6J mice given tMCAO	IP meisoindigo given before and 2 hr after reperfusion	Meisoindigo treatment reduced infarct volume, attenuated neurologic deficits, reduced cerebral edema, suppressed inflammatory response, and promoted microglia polarization to the M2 phenotype
Zheng 2019 (159)	Exosomes from LPS-stimulated macrophages induce neuroprotection and functional improvement after ischemic stroke by modulating microglial polarization	male Sprague-Dawley rats with tMCAO	IV exosomes of LPS-stimulated macrophages (LPS-Ex) given 6 hr and 24 hr after reperfusion	LPS-Ex treatment reduced infarct volume, promoted microglia polarization to the M2 phenotype, and ameliorated the post-ischemic inflammatory response
Li 2020 (160)	Edaravone-Loaded Macrophage-Derived Exosomes Enhance Neuroprotection in the Rat Permanent Middle Cerebral Artery Occlusion Model of Stroke	male Sprague-Dawley rats with permanent MCAO	IV free Edaravone (Edv) or exosomes containing Edaravone (Exo + Edv) given continuously	Edv and Exo + Edv treatments reduced mortality, Exo + Edv promoted microglia polarization to M2 phenotype
Wang 2020 (145)	FGF21 alleviates neuroinflammation following ischemic stroke by modulating the temporal and spatial dynamics of microglia/macrophages	C57BL/6 mice with tMCAO	IP rhFGF21 daily beginning 6 h post-reperfusion	rhFGF21 treatment inhibited M1 polarization of microglia, decreased pro-inflammatory cytokine expression through suppression of nuclear factor-kappa B (NF-κB) and upregulation of peroxisome proliferator-activated receptor-γ (PPAR-γ), and ameliorated behavioral neurologic deficits
Rafaelle 2021 (111)	Microglial vesicles improve post-stroke recovery by preventing immune cell senescence and favoring oligodendrogenesis	GPR17-iCreERT2:CAG-EGFP reporter mice with permanent MCAO	intracerebral infusion of IL-4 microglia-derived extracellular vesicles (EVs) given at day 14 after MCAO	IL-4 EV treatment promoted microglia polarization to the M2 phenotype, promoted OPC maturation, and enhanced neurofunctional recovery
Xu 2021 (144)	Annexin A1 protects against cerebral ischemia-reperfusion injury by modulating microglia/macrophage polarization *via* FPR2/ALX-dependent AMPK-mTOR pathway	C57BL/6J mice with tMCAO/R	IV Ac2-26 (pharmacore mimic of annexin A1) or Ac2-26 + WRW (antagonist agent) given directly after reperfusion	Ac2-26 treatment improved neurological function, reduced the volume of cerebral infarct, increased cortical cerebral blood flow, promoted the polarization of microglia/macrophages to M2 phenotype, and ameliorated BBB disruption and neuronal apoptosis

Summary of pre-clinical studies which involve microglia, monocyte, and MoDM phenotype modulation following ischemic stroke.

IP, intraperitoneal; IV, intravenous; PO, by mouth; rhFGF21, recombinant human fibroblast growth factor 21; MCAO, middle cerebral artery occlusion; tMCAO, tMCAO/R, transient middle cerebral artery occlusion/reperfusion; GMP, guanosine monophosphate; FTY720, Fingolimod; FPR2/ALX, formyl peptide receptor 2; AMPK, AMP-activated protein kinase; mTOR, mammalian target of rapamycin; KO, knockout; IL-13, interleukin 13; IL-4, interleukin 4; CCR2, C-C Motif Chemokine Receptor 2; OPC, oligodendrocyte progenitor cell.

**Table 2:** Clinical Studies Investigating Microglia Phenotype Modulation.

Study	Therapy	n	Condition	Primary Outcome	Status/Initial Results
NCT00630396	Minocycline (IV) daily	60	IS (onset < 6 hours)	Maximally Tolerated Dose	Completed Safe and well tolerated up to 10 mg/kg alone and in combination with tPA
NCT00836355	1. Minocycline (oral) 2. Enoxaparin (IV) 3. Minocycline (oral) + Enoxaparin (IV)	6	IS (onset < 6 hours)	Neuroprotection (measured by MR imaging pre and post-treatment)	Terminated
NCT00930020	Minocycline (oral) vs. placebo	139	IS (onset 3-48 hours)	Reduction of neurologic deficits and improvement of functional outcome on day 90 post-stroke	Terminated
NCT03320018	Molecular hydrogen H2 (IV or PO) + minocycline (IV or PO) vs. placebo	100	IS (onset <24 hours)	sMRSq	Unknown
NCT05121883	Edaravone Dexborneol (oral)	200	IS (onset < 48 hours)	mRS	Not yet recruiting
NCT05035953	Edaravone Dexborneol (IV) vs. placebo	200	IS (alteplase within 4.5 hours after onset)	Symptomatic ICH	Not yet recruiting
NCT04667637	Edaravone Dexborneol (IV) vs. placebo	200	Anterior IS and recanalization within 9 hours of stroke onset	mRS 0-2	Recruiting
NCT04817527	Edaravone Dexborneol (IV) vs. endovascular therapy	200	Anterior IS treated with endovascular therapy within 6-24 hours of onset	1. mRS 0-3 on day 90 2. symptomatic ICH within 48 hours 3. mTICI grade at 90 days	Not yet recruiting
NCT02430350	Compound Edaravone + Borneol vs. Edaravone(IV)	1200	IS (onset < 48 hours)	mRS ≤1 on day 90	Completed Outcomes favored Edaravone Dexborneol group, especially in female patients
NCT04984577	1. Compound Edaravone + borneol injection - Low dose - High dose 2. Edaravone injection 3. Placebo	240	IS (onset < 48 hours)	mRS ≤1 on day 90	Not yet recruiting
NCT00821821	MCI-186 (IV) vs. Placebo	36	IS (onset <24 hours)	Adverse events within 87days	Completed Safety and tolerability of MCI-186 formulation and dosing regimen was achieved
NCT01929096	1. Compound Edaravone +borneol injection - Low dose - Medium dose - High dose 2. Edaravone injection	400	IS (onset < 48 hours)	mRS score on day 90 Change from baseline NIHSS score on day 14	Completed - Edaravone Dexborneol safe and well tolerated at all doses - No improvement in functional outcomes at 90 days
NCT05024526	Edaravone dexborneol or Edaravone (IV)	80	IS	Imaging changes at 7 days	Recruiting
NCT00200356	Edaravone vs. sodium ozagrel (IV)	401	IS (onset <24 hours)	mRS of 0-1 at 3 months	Completed Edaravone at least as effective as ozagrel for treatment of acute noncardioembolic IS
NCT04950920	Y-2 tablets (Edaravone + d-borneol) vs. d-borneol (oral)	900	IS (onset ≤ 48 hours)	mRS < 1 after 90 days	Recruiting
NCT04629872	Fingolimod (oral) vs. endovascular treatment	30	Anterior IS eligible for mechanical thrombectomy within 6-24 hours of stroke onset	Collateral circulation grade compared to pre-endovascular treatment	Recruiting
NCT04718064	Fingolimod (oral) vs. placebo	20	Occlusion of M1 segment of ICA or MCA with onset <24 hours	mRS score at 90 days	Not yet recruiting
NCT04675762	Standard alteplase bridging and mechanical thrombectomy with fingolimod (oral) or placebo	118	Anterior IS eligible for alteplase and mechanical thrombectomy within 24 hours of stroke onset or awakening with stroke	Ratio of mRS score of 0-2 (%) at 90 days	Recruiting
NCT02002390	Fingolimod (oral) vs. standard of care	22	IS	Clinical improvement up to 90 days	Completed Combination therapy of fingolimod and alteplase well tolerated, attenuated reperfusion injury and improved clinical outcomes
NCT02730455	1. Natalizumab (IV) - low dose - high dose 2. placebo	277	Supratentorial IS defined by LKN ≤24 hours at treatment	Composite Global Measure of Functional Disability - Excellent Outcome at day 90	Completed Excellent outcome less likely in patients treated with natalizumab than with placebo
NCT01955707	Natalizumab (IV) vs. placebo	161	IS	Change in infarct volume from baseline	Completed No reduction in infarct growth with natalizumab but some treatment-associated benefits on functional outcomes
NCT01073007	Simvastatin (oral) vs. placebo	104	IS (onset <12 hours)	Neurological and functional outcomes at day 7/discharge or at month 3	Completed - Simvastatin + tPA combination safe in acute stroke, with low rates of bleeding complications - No statistically significant differences to show simvastatin efficacy
NCT03402204	Simvastatin 10 mg vs. Simvastatin 40 mg (oral)	64	IS (onset <24 hours)	NIHSS at 180 days	Completed No difference in clinical outcomes between high- and low-dose simvastatin
NCT00091949	Pioglitazone (oral) vs. placebo	3876	IS or TIA no less than 14 days and no more than 6 months before randomization	Recurrent Fatal or Non-fatal IS, or Fatal or Non-fatal MI up to 5 years	Completed - Pioglitazone effective for secondary prevention of IS in nondiabetic patients with insulin resistance
NCT03354429	Ticagrelor (oral)	11016	Mild-to-moderate acute noncardioembolic IS (NIHSS score ≤5) (<24 hours) or TIA	Subsequent Stroke or Death randomized from day 1 to visit 3 (day 30-34)	Completed Lower risk of death or stroke with ticagrelor-aspirin than with aspirin alone - disability did not differ significantly between the two groups - Severe bleeding more frequent with ticagrelor.
NCT04962451	Ticagrelor + ASA vs. Placebo + ASA (oral)	13000	IS (onset < 24 hours)	Subsequent Stroke or Death	Completed No results posted
NCT01994720	Ticagrelor vs. ASA (oral)	13307	IS (onset < 24 hours)	Stroke/MI/Death up to 97 days	Completed Ticagrelor not superior to aspirin in reducing the rate of stroke, MI, or death at 90 days
NCT04738097	Ticagrelor + ASA vs. Placebo + ASA (oral)	90	IS (onset < 24 hours)	IS recurrence within 3 months	Recruiting
NCT03884530	Ticagrelor vs. ASA (oral)	169	IS (onset < 9 hours) or TIA	- hemorrhagic transformation or peripheral bleeding within 48 hours of loading dose -NIHSS and mRS	Completed -better clinical outcome for ticagrelor based on NIHSS and mRS -safety profile shows ticagrelor is noninferior to aspirin

Summary of ongoing and completed clinical trials for therapies targeting specific microglia/monocyte-derived macrophage phenotypes after ischemic stroke. Minocycline has been shown to inhibit activation and proliferation of microglia and macrophages in vitro. Edaravone Dexborneol is a free radical scavenger that suppresses the inflammatory responses in activated microglia and decreases microglia-mediated inflammatory mediators. Fingolimod skews microglia toward M2 polarization after chronic cerebral hypoperfusion. Natalizumab is a monoclonal antibody against the glycoprotein α4 integrin expressed on the surface of monocytes. Simvastatin has the potential to attenuate proinflammatory mediators by controlling microglial activation and causing consequent reduction in neuroinflammatory mediators. Pioglitazone is a microglia-modulating drug which regulates anti-inflammatory activity and attenuates microglial activation through acting as an agonist of PPAR-y. Ticagrelor inhibits P2Y12-mediated microglia activation and chemotaxis.

simplified modified Rankin Scale (sMRSq), modified Rankin Scale (mRS), intracranial hemorrhage (ICH), ischemic Stroke (IS), myocardial infarction (MI), modified treatment in cerebral ischemia (mTICI), National Institutes of Health Stroke Scale (NIHSS).

Several other drugs with known immunomodulatory properties have been repurposed in an effort to downregulate stroke-induced neuroinflammation. Montelukast, an anti-asthmatic drug, is a compound which has been found to promote microglia polarization to the M2 phenotype in mice (155). Montelukast acts as an antagonist of the CysLT-1 receptor and has been found to increase the number of M2 phenotype microglia during the acute phase of stroke (155). Edaravone is a free radical scavenger used in the treatment of amyotrophic lateral sclerosis (ALS), which has been found to promote the M2 microglia activation in preclinical studies (160, 167). In a retrospective study by Enomoto et al, clinical outcomes of patients who underwent endovascular reperfusion therapy and edavarone therapy within two days of admission were compared with patients who underwent endovascular reperfusion alone. In the group that received edavarone, the authors reported significantly lower in-hospital mortality and greater functional independence at discharge (168). Additional clinical trials designed to evaluate the efficacy of Edaravone are in preparation or currently in progress (**Table 2**). Several clinical trials are investigating the efficacy of combining Edaravone with dexborneol, a food additive which has demonstrated anti-inflammatory effects in preclinical stroke models (169). Interestingly, the combined treatment of Edaravone with dexborneol has been found to have a greater benefit for female patients compared to male patients, suggesting that sex may significantly influence the efficacy of such treatments (169). Fingolimod, a sphingosine l-phosphate receptor modulator that is FDA-approved for relapsing multiple sclerosis, is another drug that was shown in preclinical studies to skew microglia towards the M2 phenotype following chronic cerebral hypoperfusion and is now being studied in several clinical trials. In one trial of 25 patients with acute hemispheric ischemic stroke, combined therapy of fingolimod and alteplase was associated with fewer circulating leukocytes, smaller infarct volumes, attenuated reperfusion injury and improved functional outcomes compared to alteplase monotherapy (170).

Exosome therapy has also been used to increase the overall number of M2 microglia in the post-stroke environment. This strategy uses extracellular vesicles (EVs) derived from multipotent mesenchymal stromal cells to deliver proteins, lipids, and/or nucleic acids. In the context of stroke, EVs have been shown to facilitate transfer of miRNAs between cells, which can influence post-transcriptional gene regulation in microglia to increase the expression of M2 markers (171). In traumatic brain injury mouse models, delivering miR-124-3p *via* EVs derived from microglia cells has been shown to promote the anti-inflammatory phenotype (172). Furthermore, infusion of EVs derived from microglia treated with IL-4 into mice following MCAO has been found to effectively promote microglia polarization to the M2 phenotype during the chronic stage of stroke. IL-4 treatment promotes functional recovery by enhancing the neuronal myelination capacity of GPR17-expressing oligodendrocyte precursor cells (111). Therefore, exosome therapy is another promising, microglia-focused therapy for ischemic stroke.

Compared to microglia-focused therapies, treatments targeting MoDMs are more limited. Preclinical studies have focused on manipulating the pro- and anti-inflammatory monocyte subtypes, as well as administering anti-inflammatory MoDMs as a form of therapy. A study by Schmidt et al. using clodronate liposomes to deplete peripheral macrophages showed no beneficial therapeutic effect after ischemic stroke in mice (153). However, when monocyte-derived macrophages were skewed to an M2 phenotype *in vitro* prior to administration into the CSF of mice after MCAO, improved cognitive and motor function was observed although there was no difference in infarct volume (101). In another preclinical study, Yang et al. demonstrated that shifting blood monocytes toward a CCR2+ proinflammatory state using remote ischemic limb conditioning (RLC) prior to stroke onset reduced brain injury and improved recovery, and that adoptive transfer of CCR2 deficient monocytes abrogated the proinflammatory shift and resulted in worse functional outcomes (157). Another study utilized hypoxic preconditioning prior to MCAO to upregulate CCL2, the receptor for CCR2, and found that it resulted in a neuroprotective phenotype with reduced infarct volume, blood-brain barrier disruption, and leukocyte migration during MCAO (58). These findings emphasize not only the importance of pro-inflammatory monocytes in stroke recovery, but also the benefit of manipulating peripheral immune cells or their chemokine signals before infiltration into the brain. There may be potential for future preventative strategies to shift monocytes to a CCR2+ subset in the acute phase of stroke or prime the microenvironment for CCR2+ cell migration earlier in the post-stroke process, such as through remote ischemic limb conditioning or hypoxic preconditioning. Furthermore, adaptive cell therapy or autologous transplantation of M2-like MoDMs into the CSF could be a promising avenue for treatment, but this will require additional studies to optimize timing of administration, dosage, and efficacy in humans.

Approaches to skewing peripheral monocytes into the M2-like phenotype have targeted factors such as PPARγ, NR4A1, and micro-RNAs (21 and 146-a) (173). Using a model of stroke-prone spontaneously hypertensive rats, Nakamura et al. demonstrated that pioglitazone, a PPARγ agonist, was protective against hypertension-induced stroke by inhibiting macrophage infiltration and suppressing the expression of inflammatory cytokines CCL2 and TNF-α (174). NR4A1, a pro-oncogenic nuclear receptor, is integral to the differentiation of classical monocytes into the M2 anti-inflammatory phenotype and may be a potential therapeutic target (175). MicroRNAs (miRNAs) have been shown to play an integral role in regulating monocyte development and function. MiRNA 146-a has been the most extensively studied and shows the largest difference in expression between classical and non-classical monocytes, with non-classical monocytes featuring higher expression. Depletion of miRNA 146-a augments the pro-inflammatory response of classical monocytes (176).

Timing of microglia and monocyte-derived macrophage migration and activity is a key consideration in developing effective therapeutic strategies. The current model is that the anti-inflammatory functions of activated microglia in the acute phase wane in the subacute phase. During this period, MoDMs fulfill an anti-inflammatory role, and the activated microglia shift toward a pro-inflammatory state. This delayed infiltration of MoDMs makes them potential candidates for immunomodulation in the subacute phase. However, our knowledge of immune cells present at each stage of ischemic stroke is currently limited to specific “snapshots” of cell populations, measured primarily through techniques such as flow cytometry and immunohistochemistry. A more fluid understanding will be necessary to target the correct cell type at the correct time (4). Future studies must also consider potential downstream effects of eliminating conventionally proinflammatory cells during each phase of stroke. As discussed, pro-inflammatory cells may have the capability to differentiate into anti-inflammatory subtypes, and therefore may be sensitive to the dynamic changes in the tissue microenvironment. Thus, nonspecific deactivation of MoDMs may decrease local tissue damage, but may also disable subsequent debris clearance and other repair mechanisms. Further elucidation of the specific contexts in which these activities occur will be critical in developing targeted immunotherapeutics.

Immunotherapies that target monocytes and MoDMs have already shown promise in neurological disorders such as encephalitis, multiple sclerosis (MS), and aneurysmal subarachnoid hemorrhage (SAH) (177–181). In a broader sense, inflammation has been shown to contribute significantly to the pathogenesis of many peripheral and central nervous system diseases, including but not limited to fibromyalgia, neuropathic pain, Alzheimer’s disease, Parkinson’s disease, and traumatic brain injury (182). A core pattern of activation by resident microglia followed by systemic myeloid cells has been shown in the aforementioned neurological diseases, with cells displaying an initial state of proinflammatory activation and a later anti-inflammatory phenotype (183). Though the inflammatory response is similar between ischemic stroke and these other neuropathologies in the sequential activation and deactivation of the innate and adaptive immune response, ischemic stroke is unique among neuropathologies in that it is caused by an acute insult that, left untreated, rapidly results in oxidative stress, apoptosis, and inflammation, leading to a massive immune response. Unlike neurodegenerative diseases, which have a more chronic, persistent immune response characterized by continuously active microglia and infiltrating leukocytes, ischemic stroke has different phases of inflammation, ranging from the acute to chronic stage. The complex orchestration of the immune response in ischemic stroke is highly dependent on the switch in activation states of microglia and MoDMs at specific times following the onset of the insult. In sum, the main differences between the way inflammation contributes to the progression of individual neurological diseases arise in a disease-specific and lesion stage-specific manner with regard to the contribution of resident versus recruited myeloid cells and their activation profiles during each stage. Ultimately, targeting the progression of neuroinflammation may be of translational benefit for a wide variety of neurological diseases.

## Conclusion

In the setting of ischemic stroke, microglia and MoDMs phagocytose cellular debris and mediate the inflammatory response by adopting pro- and anti-inflammatory activation states. These activation states are dependent upon environmental and temporal factors, and available studies suggest that inducing a phenotypic switch in microglia and MoDMs may promote stroke recovery. There are important limitations to translating this work. Markers of differentiation between microglia and monocyte-derived macrophages have historically been lacking; however, RNA sequencing data have elucidated more specific markers such as Tmem119, paving the way for targeted studies of the spatiotemporal dynamics of microglia and MoDMs in the setting of ischemic stroke. Persistent inflammation during the chronic stage of stroke is associated with impaired neurofunctional recovery, and there are no current treatments for stroke in the chronic stage beyond rehabilitation. So far, clinical trials have identified compounds which can induce anti-inflammatory microglia and MoDM activation states; however, the clinical efficacy of these compounds has yet to be confirmed. Furthermore, clinical trials have largely focused on the acute phase of stroke. To optimize neurofunctional outcomes of ischemic stroke patients, it may be necessary to apply specific immunotherapies across the spectrum of acute, subacute, and chronic inflammatory events following stroke. Future research will determine precisely which pathways should be targeted and when.

## Author Contributions

EW and KR wrote and edited the manuscript. JK was responsible for the primary literature review and edited and revised the manuscript. RX and RL edited the manuscript. CJ conceived of the manuscript and oversaw the literature review, organization and writing, and edited the final version of the manuscript. All authors contributed to the article and approved the submitted version.

## Conflict of Interest

CJ is a scientific co-founder of Egret Therapeutics with equity interests in the company and inventor on a patent filed by Johns Hopkins for using PD-1 agonists to treat cerebral vasospasm and ischemia.

The remaining authors declare that the research was conducted in the absence of any commercial or financial relationships that could be construed as a potential conflict of interest.

## Publisher’s Note

All claims expressed in this article are solely those of the authors and do not necessarily represent those of their affiliated organizations, or those of the publisher, the editors and the reviewers. Any product that may be evaluated in this article, or claim that may be made by its manufacturer, is not guaranteed or endorsed by the publisher.

## References

[1] American Heart Association. 2021 Heart Disease and Stroke Statistics Update Fact Sheet At-A-Glance (2021). Available at: https://www.heart.org/-/media/phd-files-2/science-news/2/2021-heart-and-stroke-stat-update/2021_heart_disease_and_stroke_statistics_update_fact_sheet_at_a_glance.pdf (Accessed September 7, 2021).

[2] Feigin VL Stark BA Johnson CO Roth GA Bisignano C Abady GG. Global, Regional, and National Burden of Stroke and its Risk Factors, 1990-2019: A Systematic Analysis for the Global Burden of Disease Study 2019. Lancet Neurol (2021) 20:795–820. doi: 10.1016/S1474-4422(21)00252-0 34487721 PMC8443449

[3] Virani SS Alonso A Benjamin EJ Bittencourt MS Callaway CW Carson AP. Heart Disease and Stroke Statistics—2020 Update: A Report From the American Heart Association. Circulation (2020) 141:e139–596. doi: 10.1161/CIR.0000000000000757 31992061

[4] Jin R Yang G Li G. Inflammatory Mechanisms in Ischemic Stroke: Role of Inflammatory Cells. J Leukoc Biol (2010) 87:779–89. doi: 10.1189/jlb.1109766 PMC2858674 20130219

[5] Doyle KP Quach LN Solé M Axtell RC Nguyen T Soler-Llavina GJ. B-Lymphocyte-Mediated Delayed Cognitive Impairment Following Stroke. J Neurosci (2015) 35:2133–45. doi: 10.1523/JNEUROSCI.4098-14.2015 PMC4315838 25653369

[6] Bansal S Sangha KS Khatri P. Drug Treatment of Acute Ischemic Stroke. Am J Cardiovasc Drugs (2013) 13:57–69. doi: 10.1007/s40256-013-0007-6 23381911 PMC3840541

[7] Jayaraj RL Azimullah S Beiram R Jalal FY Rosenberg GA. Neuroinflammation: Friend and Foe for Ischemic Stroke. J Neuroinflamm (2019) 16:142. doi: 10.1186/s12974-019-1516-2 PMC6617684 31291966

[8] Fagan SC Hess DC Hohnadel EJ Pollock DM Ergul A. Targets for Vascular Protection After Acute Ischemic Stroke. Stroke (2004) 35:2220–5. doi: 10.1161/01.STR.0000138023.60272.9e 15284446

[9] Gelderblom M Leypoldt F Steinbach K Behrens D Choe C-U Siler DA. Temporal and Spatial Dynamics of Cerebral Immune Cell Accumulation in Stroke. Stroke (2009) 40:1849–57. doi: 10.1161/STROKEAHA.108.534503 19265055

[10] Michaud JP Pimentel-Coelho PM Tremblay Y Rivest S. The Impact of Ly6C Low Monocytes After Cerebral Hypoxia-Ischemia in Adult Mice. J Cereb Blood Flow Metab (2014) 34:e1–9. doi: 10.1038/jcbfm.2014.80 PMC4083393 24780898

[11] Ritzel RM Patel AR Grenier JM Crapser J Verma R Jellison ER. Functional Differences Between Microglia and Monocytes After Ischemic Stroke. J Neuroinflamm (2015) 12:106. doi: 10.1186/s12974-015-0329-1 PMC4465481 26022493

[12] Miró-Mur F Pérez-de-Puig I Ferrer-Ferrer M Urra X Justicia C Chamorro A. Immature Monocytes Recruited to the Ischemic Mouse Brain Differentiate Into Macrophages With Features of Alternative Activation. Brain Behav Immun (2016) 53:18–33. doi: 10.1016/j.bbi.2015.08.010 26275369

[13] Wattananit S Tornero XD Graubardt N Memanishvili XT Monni E Tatarishvili J. Monocyte-Derived Macrophages Contribute to Spontaneous Long-Term Functional Recovery After Stroke in Mice. J Neurosci (2016) 36:4182–95. doi: 10.1523/JNEUROSCI.4317-15.2016 PMC6601783 27076418

[14] Gomez Perdiguero E Klapproth K Schulz C Busch K Azzoni E Crozet L. Tissue-Resident Macrophages Originate From Yolk-Sac-Derived Erythro-Myeloid Progenitors. Nature (2015) 518:547–51. doi: 10.1038/nature13989 PMC5997177 25470051

[15] Perry VH Nicoll JAR Holmes C. Microglia in Neurodegenerative Disease. Nat Rev Neurol (2010) 6:193–201. doi: 10.1038/nrneurol.2010.17 20234358

[16] Yang I Han SJ Kaur G Crane C Parsa AT. The Role of Microglia in Central Nervous System Immunity and Glioma Immunology. J Clin Neurosci (2010) 17:6–10. doi: 10.1016/j.jocn.2009.05.006 19926287 PMC3786731

[17] Santos EN Douglas Fields R. Regulation of Myelination by Microglia. Sci Adv (2021) 7:eabk1131. doi: 10.1126/sciadv.abk1131 34890221 PMC8664250

[18] Szalay G Martinecz B Lénárt N Környei Z Orsolits B Judák L. Microglia Protect Against Brain Injury and Their Selective Elimination Dysregulates Neuronal Network Activity After Stroke. Nat Commun (2016) 7:11499. doi: 10.1038/ncomms11499 27139776 PMC4857403

[19] Morrison HW Filosa JA. A Quantitative Spatiotemporal Analysis of Microglia Morphology During Ischemic Stroke and Reperfusion. J Neuroinflamm (2013) 10:4. doi: 10.1186/1742-2094-10-4 PMC3570327 23311642

[20] Nimmerjahn A Kirchhoff F Helmchen F. Resting Microglial Cells are Highly Dynamic Surveillants of Brain Parenchyma *In Vivo* . Science (2005) 308:1314–8. doi: 10.1126/science.1110647 15831717

[21] Gülke E Gelderblom M Magnus T. Danger Signals in Stroke and Their Role on Microglia Activation After Ischemia. Ther Adv Neurol Disord (2018) 11:1756286418774254. doi: 10.1177/1756286418774254 29854002 PMC5968660

[22] Anttila JE Whitaker KW Wires ES Harvey BK Airavaara M. Role of Microglia in Ischemic Focal Stroke and Recovery: Focus on Toll-Like Receptors. Prog Neuropsychopharmacol Biol Psychiatry (2017) 79:3–14. doi: 10.1016/j.pnpbp.2016.07.003 27389423 PMC5214845

[23] Wang YC Lin S Yang QW. Toll-Like Receptors in Cerebral Ischemic Inflammatory Injury. J Neuroinflamm (2011) 8:134. doi: 10.1186/1742-2094-8-134 PMC3198933 21982558

[24] Galloway DA Phillips AEM Owen DRJ Moore CS. Phagocytosis in the Brain: Homeostasis and Disease. Front Immunol (2019) 10:790. doi: 10.3389/fimmu.2019.00790 31040847 PMC6477030

[25] Das R Chinnathambi S. Actin-Mediated Microglial Chemotaxis *via* G-Protein Coupled Purinergic Receptor in Alzheimer’s Disease. Neuroscience (2020) 448:325–36. doi: 10.1016/j.neuroscience.2020.09.024 32941933

[26] Bachiller S Jiménez-Ferrer I Paulus A Yang Y Swanberg M Deierborg T. Microglia in Neurological Diseases: A Road Map to Brain-Disease Dependent-Inflammatory Response. Front Cell Neurosci (2018) 12:488. doi: 10.3389/fncel.2018.00488 30618635 PMC6305407

[27] Bartels T de Schepper S Hong S. Microglia Modulate Neurodegeneration in Alzheimer’s and Parkinson’s Diseases. Science (2020) 370:66–9. doi: 10.1126/science.abb8587 33004513

[28] Christopher E Loan JJM Samarasekera N McDade K Rose J Barrington J. Nrf2 Activation in the Human Brain After Stroke Due to Supratentorial Intracerebral Haemorrhage: A Case-Control Study. BMJ Neurol Open (2022) 4:e000238. doi: 10.1136/bmjno-2021-000238 PMC8860052 35265844

[29] Wang Y Huang Y Xu Y Ruan W Wang H Zhang Y. A Dual AMPK/Nrf2 Activator Reduces Brain Inflammation After Stroke by Enhancing Microglia M2 Polarization. Antioxid Redox Signal (2018) 28:141–63. doi: 10.1089/ars.2017.7003 28747068

[30] Lei X Li H Li M Dong Q Zhao H Zhang Z. The Novel Nrf2 Activator CDDO-EA Attenuates Cerebral Ischemic Injury by Promoting Microglia/Macrophage Polarization Toward M2 Phenotype in Mice. CNS Neurosci Ther (2021) 27:82–91. doi: 10.1111/cns.13496 33280237 PMC7804925

[31] Ślusarczyk J Trojan E Glombik K Piotrowska A Budziszewska B Kubera M. Targeting the NLRP3 Inflammasome-Related Pathways *via* Tianeptine Treatment-Suppressed Microglia Polarization to the M1 Phenotype in Lipopolysaccharide-Stimulated Cultures. Int J Mol Sci (2018) 19:1965. doi: 10.3390/ijms19071965 PMC6073715 29976873

[32] Aryanpour R Pasbakhsh P Zibara K Namjoo Z Beigi Boroujeni F Shahbeigi S. Progesterone Therapy Induces an M1 to M2 Switch in Microglia Phenotype and Suppresses NLRP3 Inflammasome in a Cuprizone-Induced Demyelination Mouse Model. Int Immunopharmacol (2017) 51:131–9. doi: 10.1016/j.intimp.2017.08.007 28830026

[33] Masuda T Sankowski R Staszewski O Prinz M. Microglia Heterogeneity in the Single-Cell Era. Cell Rep (2020) 30:1271–81. doi: 10.1016/j.celrep.2020.01.010 32023447

[34] Franco R Fernandez-Suarez D. Alternatively Activated Microglia and Macrophages in the Central Nervous System. Prog Neurobiol (2015) 131:65–86. doi: 10.1016/j.pneurobio.2015.05.003 26067058

[35] Schulz C Perdiguero EG Chorro L Szabo-Rogers H Cagnard N Kierdorf K. A Lineage of Myeloid Cells Independent of Myb and Hematopoietic Stem Cells. Science (2012) 336:86–90. doi: 10.1126/science.1219179 22442384

[36] Pittet MJ Nahrendorf M Swirski FK. The Journey From Stem Cell to Macrophage. Ann N Y Acad Sci (2014) 1319:1–18. doi: 10.1111/nyas.12393 24673186 PMC4074243

[37] Mosser DM Hamidzadeh K Goncalves R. Macrophages and the Maintenance of Homeostasis. Cell Mol Immunol (2021) 18:579–87. doi: 10.1038/s41423-020-00541-3 PMC7491045 32934339

[38] Sica A Mantovani A. Macrophage Plasticity and Polarization: *In Vivo* Veritas. J Clin Invest (2012) 122:787–95. doi: 10.1172/JCI59643 PMC3287223 22378047

[39] Pollard JW. Trophic Macrophages in Development and Disease. Nat Rev Immunol (2009) 9:259–70. doi: 10.1038/nri2528 PMC3648866 19282852

[40] Murray PJ Wynn TA. Protective and Pathogenic Functions of Macrophage Subsets. Nat Rev Immunol (2011) 11:723–37. doi: 10.1038/nri3073 PMC3422549 21997792

[41] Li Q Barres BA. Microglia and Macrophages in Brain Homeostasis and Disease. Nat Rev Immunol (2018) 18:225–42. doi: 10.1038/nri.2017.125 29151590

[42] van Wageningen TA Vlaar E Kooij G Jongenelen CAM Geurts JJG van Dam AM. Regulation of Microglial TMEM119 and P2RY12 Immunoreactivity in Multiple Sclerosis White and Grey Matter Lesions is Dependent on Their Inflammatory Environment. Acta Neuropathol Commun (2019) 7:206. doi: 10.1186/s40478-019-0850-z 31829283 PMC6907356

[43] Amici SA Dong J Guerau-de-Arellano M. Molecular Mechanisms Modulating the Phenotype of Macrophages and Microglia. Front Immunol (2017) 8:1520. doi: 10.3389/fimmu.2017.01520 29176977 PMC5686097

[44] Ziegler-Heitbrock L Ancuta P Crowe S Dalod M Grau V Hart DN. Nomenclature of Monocytes and Dendritic Cells in Blood. Blood (2010) 116:e74–80. doi: 10.1182/blood-2010-02-258558 20628149

[45] Grage-Griebenow E Zawatzky R Kahlert H Brade L Flad H-D Ernst M. Identification of a Novel Dendritic Cell-Like Subset of CD64 + /CD16 + Blood Monocytes. Eur J Immunol (2001) 31:48–56. doi: 10.1002/1521-4141(200101)31:1<48::aid-immu48>3.0.co;2-5 11169437

[46] Grage-Griebenow E Flad H-D Ernst M. Heterogeneity of Human Peripheral Blood Monocyte Subsets. J Leukoc Biol (2001) 69:11–20. doi: 10.1189/jlb.69.1.11 11200054

[47] Auffray C Sieweke MH Geissmann F. Blood Monocytes: Development, Heterogeneity, and Relationship With Dendritic Cells. Annu Rev Immunol (2009) 27:669–92. doi: 10.1146/annurev.immunol.021908.132557 19132917

[48] Kapellos TS Bonaguro L Gemünd I Reusch N Saglam A Hinkley ER. Human Monocyte Subsets and Phenotypes in Major Chronic Inflammatory Diseases. Front Immunol (2019) 10:2035. doi: 10.3389/fimmu.2019.02035 31543877 PMC6728754

[49] Shi C Pamer EG. Monocyte Recruitment During Infection and Inflammation. Nat Rev Immunol (2011) 11:762–74. doi: 10.1038/nri3070 PMC3947780 21984070

[50] Geissmann F Jung S Littman DR. Blood Monocytes Consist of Two Principal Subsets With Distinct Migratory Properties. Immunity (2003) 19:71–82. doi: 10.1016/s1074-7613(03)00174-2 12871640

[51] Hanna RN Carlin LM Hubbeling HG Nackiewicz D Green AM Punt JA. The Transcription Factor NR4A1 (Nur77) Controls Bone Marrow Differentiation and the Survival of Ly6C- Monocytes. Nat Immunol (2011) 12:778–85. doi: 10.1038/ni.2063 PMC3324395 21725321

[52] Ingersoll MA Spanbroek R Lottaz C Gautier EL Frankenberger M Hoffmann R. Comparison of Gene Expression Profiles Between Human and Mouse Monocyte Subsets. Blood (2010) 115:e10–9. doi: 10.1182/blood-2009-07-235028 PMC2810986 19965649

[53] Ginhoux F Jung S. Monocytes and Macrophages: Developmental Pathways and Tissue Homeostasis. Nat Rev Immunol (2014) 14:392–404. doi: 10.1038/nri3671 24854589

[54] Thériault P Elali A Rivest S. The Dynamics of Monocytes and Microglia in Alzheimer’s Disease. Alzheimers Res Ther (2015) 7:41. doi: 10.1186/s13195-015-0125-2 25878730 PMC4397873

[55] Palframan RT Jung S Cheng G Weninger W Luo Y Dorf M. Inflammatory Chemokine Transport and Presentation in HEV: A Remote Control Mechanism for Monocyte Recruitment to Lymph Nodes in Inflamed Tissues. J Exp Med (2001) 194:1361–73. doi: 10.1084/jem.194.9.1361 PMC2195988 11696600

[56] Passlick B Flieger D Ziegler-Heitbrock HW. Identification and Characterization of a Novel Monocyte Subpopulation in Human Peripheral Blood. Blood (1989) 74:2527–34. doi: 10.1182/blood.V74.7.2527.2527 2478233

[57] Auffray C Fogg D Garfa M Elain G Join-Lambert O Kayal S. Monitoring of Blood Vessels and Tissues by a Population of Monocytes With Patrolling Behavior. Science (2007) 317:666–70. doi: 10.1126/science.1142883 17673663

[58] Stowe AM Wacker BK Cravens PD Perfater JL Li MK Hu R. CCL2 Upregulation Triggers Hypoxic Preconditioning-Induced Protection From Stroke. J Neuroinflamm (2012) 9:33. doi: 10.1186/1742-2094-9-33 PMC3298779 22340958

[59] Tsou CL Peters W Si Y Slaymaker S Aslanian AM Weisberg SP. Critical Roles for CCR2 and MCP-3 in Monocyte Mobilization From Bone Marrow and Recruitment to Inflammatory Sites. J Clin Invest (2007) 117:902–9. doi: 10.1172/JCI29919 PMC1810572 17364026

[60] Kaufmann A Salentin R Gemsa D Sprenger H. Increase of CCR1 and CCR5 Expression and Enhanced Functional Response to MIP-1α During Differentiation of Human Monocytes to Macrophages. J Leukoc Biol (2001) 69:248–52. doi: 10.1189/jlb.69.2.248 11272275

[61] Charo IF Ransohoff RM. The Many Roles of Chemokines and Chemokine Receptors in Inflammation. N Engl J Med (2006) . 354:610–21. doi: 10.1056/NEJMra052723 16467548

[62] Tacke F Alvarez D Kaplan TJ Jakubzick C Spanbroek R Llodra J. Monocyte Subsets Differentially Employ CCR2, CCR5, and CX3CR1 to Accumulate Within Atherosclerotic Plaques. J Clin Invest (2007) 117:185–94. doi: 10.1172/JCI28549 PMC1716202 17200718

[63] Balashov KE Rottman JB Weiner HL Hancock WW. CCR5 and CXCR3 T Cells are Increased in Multiple Sclerosis and Their Ligands MIP-1 and IP-10 are Expressed in Demyelinating Brain Lesions. Proc Natl Acad Sci U S A (1999) 96:6873–8. doi: 10.1073/pnas.96.12.6873 PMC22009 10359806

[64] Ravindran R Rusch L Itano A Jenkins MK Mcsorley SJ. CCR6-Dependent Recruitment of Blood Phagocytes is Necessary for Rapid CD4 T Cell Responses to Local Bacterial Infection. Proc Natl Acad Sci U S A (2007) 104:12075–80. doi: 10.1073/pnas.0701363104 PMC1907313 17615242

[65] Qu C Edwards EW Tacke F Angeli V Llodrá J Sanchez-Schmitz G. Role of CCR8 and Other Chemokine Pathways in the Migration of Monocyte-Derived Dendritic Cells to Lymph Nodes. J Exp Med (2004) 200:1231–41. doi: 10.1084/jem.20032152 PMC2211916 15534368

[66] Tacke F. Functional Role of Intrahepatic Monocyte Subsets for the Progression of Liver Inflammation and Liver Fibrosis *In Vivo* . Fibrogenesis Tissue Repair (2012) 5:S27. doi: 10.1186/1755-1536-5-S1-S27 23259611 PMC3368797

[67] Rosen H Gordon S. Monoclonal Antibody to the Murine Type 3 Complement Receptor Inhibits Adhesion of Myelomonocytic Cells *In Vitro* and Inflammatory Cell Recruitment *In Vivo* . J Exp Med (1987) 166:1685–701. doi: 10.1084/jem.166.6.1685 PMC2188801 2445894

[68] León B Ardavín C. Monocyte Migration to Inflamed Skin and Lymph Nodes is Differentially Controlled by L-Selectin and PSGL-1. Blood (2008) 111:3126–30. doi: 10.1182/blood-2007-07-100610 18184867

[69] Sun D Zhang M Sun P Liu G Strickland AB Chen Y. VCAM1/VLA4 Interaction Mediates Ly6Clow Monocyte Recruitment to the Brain in a TNFR Signaling Dependent Manner During Fungal Infection. PLoS Pathog (2020) 16:e1008361. doi: 10.1371/journal.ppat.1008361 32101593 PMC7062284

[70] Tedder TF Steeber DA Pizcueta P. L-Selectin-Deficient Mice Have Impaired Leukocyte Recruitment Into Inflammatory Sites. J Exp Med (1995) 181:2259–64. doi: 10.1084/jem.181.6.2259 PMC2192046 7539045

[71] Yang J Zhang L Yu C Yang XF Wang H. Monocyte and Macrophage Differentiation: Circulation Inflammatory Monocyte as Biomarker for Inflammatory Diseases. biomark Res (2014) 2:1. doi: 10.1186/2050-7771-2-1 24398220 PMC3892095

[72] Gliem M Mausberg AK Lee JI Simiantonakis I van Rooijen N Hartung HP. Macrophages Prevent Hemorrhagic Infarct Transformation in Murine Stroke Models. Ann Neurol (2012) 71:743–52. doi: 10.1002/ana.23529 22718543

[73] Xue J Schmidt SV Sander J Draffehn A Krebs W Quester I. Transcriptome-Based Network Analysis Reveals a Spectrum Model of Human Macrophage Activation. Immunity (2014) 40:274–88. doi: 10.1016/j.immuni.2014.01.006 PMC3991396 24530056

[74] Schulz D Severin Y Zanotelli VRT Bodenmiller B. In-Depth Characterization of Monocyte-Derived Macrophages Using a Mass Cytometry-Based Phagocytosis Assay. Sci Rep (2019) 9:1925. doi: 10.1038/s41598-018-38127-9 30760760 PMC6374473

[75] Garcia-Bonilla L Faraco G Moore J Murphy M Racchumi G Srinivasan J. Spatio-Temporal Profile, Phenotypic Diversity, and Fate of Recruited Monocytes Into the Post-Ischemic Brain. J Neuroinflamm (2016) 13:285. doi: 10.1186/s12974-016-0750-0 PMC5097435 27814740

[76] Belov Kirdajova D Kriska J Tureckova J Anderova M. Ischemia-Triggered Glutamate Excitotoxicity From the Perspective of Glial Cells. Front Cell Neurosci (2020) 14:51. doi: 10.3389/fncel.2020.00051 32265656 PMC7098326

[77] Yanuck SF. Microglial Phagocytosis of Neurons: Diminishing Neuronal Loss in Traumatic, Infectious, Inflammatory, and Autoimmune CNS Disorders. Front Psychiatry (2019) 10:712. doi: 10.3389/fpsyt.2019.00712 31632307 PMC6786049

[78] Yu L Su X Li S Zhao F Mu D Qu Y. Microglia and Their Promising Role in Ischemic Brain Injuries: An Update. Front Cell Neurosci (2020) 14:211. doi: 10.3389/fncel.2020.00211 32754016 PMC7365911

[79] Ito D Tanaka K Suzuki S Dembo T Fukuuchi Y. Enhanced Expression of Iba1, Ionized Calcium-Binding Adapter Molecule 1, After Transient Focal Cerebral Ischemia in Rat Brain. Stroke (2001) 32:1208–15. doi: 10.1161/01.str.32.5.1208 11340235

[80] Gelosa P Lecca D Fumagalli M Wypych D Pignieri A Cimino M. Microglia is a Key Player in the Reduction of Stroke Damage Promoted by the New Antithrombotic Agent Ticagrelor. J Cereb Blood Flow Metab (2014) 34:979–88. doi: 10.1038/jcbfm.2014.45 PMC4050242 24643079

[81] Fan Y Xie L Chung CY. Signaling Pathways Controlling Microglia Chemotaxis. Mol Cells (2017) 40:163–8. doi: 10.14348/molcells.2017.0011 PMC5386953 28301917

[82] Tang Y Le W. Differential Roles of M1 and M2 Microglia in Neurodegenerative Diseases. Mol Neurobiol (2016) 53:1181–94. doi: 10.1007/s12035-014-9070-5 25598354

[83] Jurga AM Paleczna M Kuter KZ. Overview of General and Discriminating Markers of Differential Microglia Phenotypes. Front Cell Neurosci (2020) 14:198. doi: 10.3389/fncel.2020.00198 32848611 PMC7424058

[84] Taylor RA Sansing LH. Microglial Responses After Ischemic Stroke and Intracerebral Hemorrhage. Clin Dev Immunol (2013) 2013:746068. doi: 10.1155/2013/746068 24223607 PMC3810327

[85] Rupalla K Allegrini PR Sauer D Wiessner C. Time Course of Microglia Activation and Apoptosis in Various Brain Regions After Permanent Focal Cerebral Ischemia in Mice. Acta Neuropathol (1998) 96:172–8. doi: 10.1007/s004010050878 9705133

[86] Koizumi T Kerkhofs D Mizuno T Steinbusch HWM Foulquier S. Vessel-Associated Immune Cells in Cerebrovascular Diseases: From Perivascular Macrophages to Vessel-Associated Microglia. Front Neurosci (2019) 13:1291. doi: 10.3389/fnins.2019.01291 31866808 PMC6904330

[87] Haruwaka K Ikegami A Tachibana Y Ohno N Konishi H Hashimoto A. Dual Microglia Effects on Blood Brain Barrier Permeability Induced by Systemic Inflammation. Nat Commun (2019) 10:5816. doi: 10.1038/s41467-019-13812-z 31862977 PMC6925219

[88] Jolivel V Bicker F Binamé F Ploen R Keller S Gollan R. Perivascular Microglia Promote Blood Vessel Disintegration in the Ischemic Penumbra. Acta Neuropathol (2015) 129:279–95. doi: 10.1007/s00401-014-1372-1 25500713

[89] Murphy SJ McCullough LD Smith JM. Stroke in the Female: Role of Biological Sex and Estrogen. ILAR J (2004) 45:147–59. doi: 10.1093/ilar.45.2.147 15111734

[90] Spychala MS Honarpisheh P McCullough LD. Sex Differences in Neuroinflammation and Neuroprotection in Ischemic Stroke. J Neurosci Res (2017) 95:462–71. doi: 10.1002/jnr.23962 PMC5217708 27870410

[91] Villa A Gelosa P Castiglioni L Cimino M Rizzi N Pepe G. Sex-Specific Features of Microglia From Adult Mice. Cell Rep (2018) 23:3501–11. doi: 10.1016/j.celrep.2018.05.048 PMC6024879 29924994

[92] Zhao SC Wang C Xu H Wu WQ Chu ZH Ma LS. Age-Related Differences in Interferon Regulatory Factor-4 and -5 Signaling in Ischemic Brains of Mice. Acta Pharmacol Sin (2017) 38:1425–34. doi: 10.1038/aps.2017.122 PMC5672072 28905935

[93] Fenn AM Henry CJ Huang Y Dugan A Godbout JP. Lipopolysaccharide-Induced Interleukin (IL)-4 Receptor-α Expression and Corresponding Sensitivity to the M2 Promoting Effects of IL-4 are Impaired in Microglia of Aged Mice. Brain Behav Immun (2012) 26:766–77. doi: 10.1016/j.bbi.2011.10.003 PMC3288757 22024136

[94] Mildner A MacK M Schmidt H Brück W Djukic M Zabel MD. CCR2+Ly-6Chi Monocytes are Crucial for the Effector Phase of Autoimmunity in the Central Nervous System. Brain (2009) 132:2487–500. doi: 10.1093/brain/awp144 19531531

[95] Serbina Nv Pamer EG. Monocyte Emigration From Bone Marrow During Bacterial Infection Requires Signals Mediated by Chemokine Receptor CCR2. Nat Immunol (2006) 7:311–7. doi: 10.1038/ni1309 16462739

[96] Kim E Yang J Beltran CD Cho S. Role of Spleen-Derived Monocytes/Macrophages in Acute Ischemic Brain Injury. J Cereb Blood Flow Metab (2014) 34:1411–9. doi: 10.1038/jcbfm.2014.101 PMC4126087 24865998

[97] Nadareishvili Z Simpkins AN Hitomi E Reyes D Leigh R. Post-Stroke Blood-Brain Barrier Disruption and Poor Functional Outcome in Patients Receiving Thrombolytic Therapy. Cerebrovasc Dis (2019) 47:135–42. doi: 10.1159/000499666 PMC6610790 30970357

[98] Yang Y Rosenberg GA. Blood-Brain Barrier Breakdown in Acute and Chronic Cerebrovascular Disease. Stroke (2011) 42:3323–8. doi: 10.1161/STROKEAHA.110.608257 PMC3584169 21940972

[99] Su Y Gao J Kaur P Wang Z. Neutrophils and Macrophages as Targets for Development of Nanotherapeutics in Inflammatory Diseases. Pharmaceutics (2020) 12:1222. doi: 10.3390/pharmaceutics12121222 PMC7766591 33348630

[100] Yilmaz G Granger DN. Cell Adhesion Molecules and Ischemic Stroke. Neurol Res (2008) 30:783–93. doi: 10.1179/174313208X341085 PMC2748428 18826804

[101] Ge R Tornero D Hirota M Monni E Laterza C Lindvall O. Choroid Plexus-Cerebrospinal Fluid Route for Monocyte-Derived Macrophages After Stroke. J Neuroinflamm (2017) 14:153. doi: 10.1186/s12974-017-0909-3 PMC5534106 28754163

[102] Werner Y Mass E Ashok Kumar P Ulas T Handler K Horne A. Cxcr4 Distinguishes HSC-Derived Monocytes From Microglia and Reveals Monocyte Immune Responses to Experimental Stroke. Nat Neurosci (2020) 23:351–62. doi: 10.1038/s41593-020-0585-y PMC7523735 32042176

[103] Perego C Fumagalli S De Simoni MG. Temporal Pattern of Expression and Colocalization of Microglia/Macrophage Phenotype Markers Following Brain Ischemic Injury in Mice. J Neuroinflamm (2011) 8:174. doi: 10.1186/1742-2094-8-174 PMC3251548 22152337

[104] Hu X Li P Guo Y Wang H Leak RK Chen S. Microglia/macrophage Polarization Dynamics Reveal Novel Mechanism of Injury Expansion After Focal Cerebral Ischemia. Stroke (2012) 43:3063–70. doi: 10.1161/STROKEAHA.112.659656 22933588

[105] Cai W Yang T Liu H Han L Zhang K Hu X. Peroxisome Proliferator-Activated Receptor Gamma (PPARgamma): A Master Gatekeeper in CNS Injury and Repair. Prog Neurobiol (2018) 163-164:27–58. doi: 10.1016/j.pneurobio.2017.10.002 29032144 PMC6037317

[106] Mori S Maher P Conti B. Neuroimmunology of the Interleukins 13 and 4. Brain Sci (2016) 6:18. doi: 10.3390/brainsci6020018 PMC4931495 27304970

[107] Lyu J Xie D Bhatia TN Leak RK Hu X Jiang X. Microglial/Macrophage Polarization and Function in Brain Injury and Repair After Stroke. CNS Neurosci Ther (2021) 27:515–27. doi: 10.1111/cns.13620 PMC8025652 33650313

[108] Lian L Zhang Y Liu L Yang L Cai Y Zhang J. Neuroinflammation in Ischemic Stroke: Focus on MicroRNA-Mediated Polarization of Microglia. Front Mol Neurosci (2020) 13:612439110. doi: 10.3389/fnmol.2020.612439110 PMC7817943 33488360

[109] Jiang CT Wu WF Deng YH Ge JW. Modulators of Microglia Activation and Polarization in Ischemic Stroke (Review). Mol Med Rep (2020) 21:2006–18. doi: 10.3892/mmr.2020.11003 PMC7115206 32323760

[110] Gaire BP. Microglia as the Critical Regulators of Neuroprotection and Functional Recovery in Cerebral Ischemia. Cell Mol Neurobiol (2021) 1–21. doi: 10.1007/s10571-021-01145-9 PMC11421653 34460037

[111] Raffaele S Gelosa P Bonfanti E Lombardi M Castiglioni L Cimino M. Microglial Vesicles Improve Post-Stroke Recovery by Preventing Immune Cell Senescence and Favoring Oligodendrogenesis. Mol Ther (2021) 29:1439–58. doi: 10.1016/j.ymthe.2020.12.009 PMC8058432 33309882

[112] Liu T Zhang L Joo D Sun SC. NF-kappaB Signaling in Inflammation. Signal Transduct Target Ther (2017) 2:17023. doi: 10.1038/sigtrans.2017.23 29158945 PMC5661633

[113] Yu F Huang T Ran Y Li D Ye L Tian G. New Insights Into the Roles of Microglial Regulation in Brain Plasticity-Dependent Stroke Recovery. Front Cell Neurosci (2021) 15:727899. doi: 10.3389/fncel.2021.727899 34421544 PMC8374071

[114] Gerhard A Schwarz J Myers R Wise R Banati RB. Evolution of Microglial Activation in Patients After Ischemic Stroke: A [11C](R)-PK11195 PET Study. Neuroimage (2005) 24:591–5. doi: 10.1016/j.neuroimage.2004.09.034 15627603

[115] Dong R Huang R Wang J Liu H Xu Z. Effects of Microglial Activation and Polarization on Brain Injury After Stroke. Front Neurol (2021) 12:620948. doi: 10.3389/fneur.2021.620948 34276530 PMC8280287

[116] ElAli A LeBlanc NJ. The Role of Monocytes in Ischemic Stroke Pathobiology: New Avenues to Explore. Front Aging Neurosci (2016) 8:29. doi: 10.3389/fnagi.2016.00029 26941641 PMC4761876

[117] Kaito M Araya S-I Gondo Y Fujita M Minato N Nakanishi M. Relevance of Distinct Monocyte Subsets to Clinical Course of Ischemic Stroke Patients. PLoS One (2013) 8:e69409. doi: 10.1371/journal.pone.0069409 23936327 PMC3732285

[118] Cherry JD Olschowka JA O’Banion MK. Neuroinflammation and M2 Microglia: The Good, the Bad, and the Inflamed. J Neuroinflamm (2014) 11:98. doi: 10.1186/1742-2094-11-98 PMC4060849 24889886

[119] Hou D Wang C Ye X Zhong P Wu D. Persistent Inflammation Worsens Short-Term Outcomes in Massive Stroke Patients. BMC Neurol (2021) 21:62. doi: 10.1186/s12883-021-02097-9 33568099 PMC7874622

[120] Wierońska JM Cieslik P Kalinowski L. Nitric Oxide-Dependent Pathways as Critical Factors in the Consequences and Recovery After Brain Ischemic Hypoxia. Biomolecules (2021) 11:1097. doi: 10.3390/biom11081097 34439764 PMC8392725

[121] Bennett ML Bennett FC Liddelow SA Ajami B Zamanian JL Fernhoff NB. New Tools for Studying Microglia in the Mouse and Human CNS. Proc Natl Acad Sci U S A (2016) 113:E1738–46. doi: 10.1073/pnas.1525528113 PMC4812770 26884166

[122] Breckwoldt MO Chen JW Stangenberg L Aikawa E Rodriguez E Qiu S. Tracking the Inflammatory Response in Stroke *In Vivo* by Sensing the Enzyme Myeloperoxidase. Proc Natl Acad Sci U S A (2008) 105:18584–9. doi: 10.1073/pnas.0803945105 PMC2587593 19011099

[123] Tanaka R Komine-Kobayashi M Mochizuki H Yamada M Furuya T Migita M. Migration of Enhanced Green Fluorescent Protein Expressing Bone Marrow-Derived Microglia/Macrophage Into the Mouse Brain Following Permanent Focal Ischemia. Neuroscience (2003) 117:531–9. doi: 10.1016/s0306-4522(02)00954-5 12617960

[124] Schilling M Besselmann M Muller M Strecker JK Ringelstein EB Kiefer R. Predominant Phagocytic Activity of Resident Microglia Over Hematogenous Macrophages Following Transient Focal Cerebral Ischemia: An Investigation Using Green Fluorescent Protein Transgenic Bone Marrow Chimeric Mice. Exp Neurol (2005) 196:290–7. doi: 10.1016/j.expneurol.2005.08.004 16153641

[125] Schilling M Strecker JK Schabitz WR Ringelstein EB Kiefer R. Effects of Monocyte Chemoattractant Protein 1 on Blood-Borne Cell Recruitment After Transient Focal Cerebral Ischemia in Mice. Neuroscience (2009) 161:806–12. doi: 10.1016/j.neuroscience.2009.04.025 19374937

[126] Petry KG Boiziau C Dousset V Brochet B. Magnetic Resonance Imaging of Human Brain Macrophage Infiltration. Neurotherapeutics (2007) 4:434–42. doi: 10.1016/j.nurt.2007.05.005 PMC7479730 17599709

[127] Crane MJ Daley JM van Houtte O Brancato SK Henry WL Jr. Albina JE. The Monocyte to Macrophage Transition in the Murine Sterile Wound. PLoS One (2014) 9:e86660. doi: 10.1371/journal.pone.0086660 24466192 PMC3899284

[128] Sachet M Liang YY Oehler R. The Immune Response to Secondary Necrotic Cells. Apoptosis (2017) 22:1189–204. doi: 10.1007/s10495-017-1413-z PMC5630647 28861714

[129] Greenhalgh AD Zarruk JG Healy LM Baskar Jesudasan SJ Jhelum P Salmon CK. Peripherally Derived Macrophages Modulate Microglial Function to Reduce Inflammation After CNS Injury. PLoS Biol (2018) 16:e2005264. doi: 10.1371/journal.pbio.2005264 30332405 PMC6205650

[130] Kolosowska N Keuters MH Wojciechowski S Keksa-Goldsteine V Laine M Malm T. Peripheral Administration of IL-13 Induces Anti-Inflammatory Microglial/Macrophage Responses and Provides Neuroprotection in Ischemic Stroke. Neurotherapeutics (2019) 16:1304–19. doi: 10.1007/s13311-019-00761-0 PMC6985054 31372938

[131] Zhang W Zhao J Wang R Jiang M Ye Q Smith AD. Macrophages Reprogram After Ischemic Stroke and Promote Efferocytosis and Inflammation Resolution in the Mouse Brain. CNS Neurosci Ther (2019) 25:1329–42. doi: 10.1111/cns.13256 PMC6887920 31697040

[132] Shechter R London A Varol C Raposo C Cusimano M Yovel G. Infiltrating Blood-Derived Macrophages are Vital Cells Playing an Anti-Inflammatory Role in Recovery From Spinal Cord Injury in Mice. PLoS Med (2009) 6:e1000113. doi: 10.1371/journal.pmed.1000113 19636355 PMC2707628

[133] Fang W Zhai X Han D Xiong X Wang T Zeng X. CCR2-Dependent Monocytes/Macrophages Exacerbate Acute Brain Injury But Promote Functional Recovery After Ischemic Stroke in Mice. Theranostics (2018) 8:3530–43. doi: 10.7150/thno.24475 PMC6037034 30026864

[134] Ma Y Li Y Jiang L Wang L Jiang Z Wang Y. Macrophage Depletion Reduced Brain Injury Following Middle Cerebral Artery Occlusion in Mice. J Neuroinflamm (2016) 13:38. doi: 10.1186/s12974-016-0504-z PMC4752808 26873581

[135] Hammond MD Taylor RA Mullen MT Ai Y Aguila HL Mack M. CCR2+ Ly6C(hi) Inflammatory Monocyte Recruitment Exacerbates Acute Disability Following Intracerebral Hemorrhage. J Neurosci (2014) 34:3901–9. doi: 10.1523/JNEUROSCI.4070-13.2014 PMC3951693 24623768

[136] Kanazawa M Takahashi T Ishikawa M Onodera O Shimohata T Del Zoppo GJ. Angiogenesis in the Ischemic Core: A Potential Treatment Target? J Cereb Blood Flow Metab (2019) 39:753–69. doi: 10.1177/0271678X19834158 PMC6501515 30841779

[137] Jung H-Y. Rehabilitation in Subacute and Chronic Stage After Stroke. In: Stroke Revisited: Diagnosis and Treatment of Ischemic Stroke. Singapore: Springer (2017). p. 351–60.

[138] Szelenberger R Kostka J Saluk-Bijak J Miller E. Pharmacological Interventions and Rehabilitation Approach for Enhancing Brain Self-Repair and Stroke Recovery. Curr Neuropharmacol (2020) 18:51–64. doi: 10.2174/1570159X17666190726104139 31362657 PMC7327936

[139] Rogalewski A Schäbitz WR. Stroke Recovery Enhancing Therapies: Lessons From Recent Clinical Trials. Neural Regener Res (2022) 17:717–20. doi: 10.4103/1673-5374.314287 PMC8530130 34472456

[140] Qin C Fan WH Liu Q Shang K Murugan M Wu LJ. Fingolimod Protects Against Ischemic White Matter Damage by Modulating Microglia Toward M2 Polarization *via* STAT3 Pathway. Stroke (2017) 48:3336–46. doi: 10.1161/STROKEAHA.117.018505 PMC5728178 29114096

[141] Chu X Cao L Yu Z Xin D Li T Ma W. Hydrogen-Rich Saline Promotes Microglia M2 Polarization and Complement-Mediated Synapse Loss to Restore Behavioral Deficits Following Hypoxia-Ischemic in Neonatal Mice *via* AMPK Activation. J Neuroinflamm (2019) 16:104. doi: 10.1186/s12974-019-1488-2 PMC6525972 31103039

[142] Li L Gan H Jin H Fang Y Yang Y Zhang J. Astragaloside IV Promotes Microglia/Macrophages M2 Polarization and Enhances Neurogenesis and Angiogenesis Through PPARgamma Pathway After Cerebral Ischemia/Reperfusion Injury in Rats. Int Immunopharmacol (2021) 92:107335. doi: 10.1016/j.intimp.2020.107335 33429332

[143] Li F Zhao H Han Z Wang R Tao Z Fan Z. Xuesaitong May Protect Against Ischemic Stroke by Modulating Microglial Phenotypes and Inhibiting Neuronal Cell Apoptosis *via* the STAT3 Signaling Pathway. CNS Neurol Disord Drug Targets (2019) 18:115–23. doi: 10.2174/1871527317666181114140340 30426907

[144] Xu X Gao W Li L Hao J Yang B Wang T. Annexin A1 Protects Against Cerebral Ischemia-Reperfusion Injury by Modulating Microglia/Macrophage Polarization *via* FPR2/ALX-Dependent AMPK-mTOR Pathway. J Neuroinflamm (2021) 18:119. doi: 10.1186/s12974-021-02174-3 PMC8140477 34022892

[145] Wang D Liu F Zhu L Lin P Han F Wang X. FGF21 Alleviates Neuroinflammation Following Ischemic Stroke by Modulating the Temporal and Spatial Dynamics of Microglia/Macrophages. J Neuroinflamm (2020) 17:257. doi: 10.1186/s12974-020-01921-2 PMC7457364 32867781

[146] Blacker DJ Prentice D Alvaro A Bates TR Bynevelt M Kelly A. Reducing Haemorrhagic Transformation After Thrombolysis for Stroke: A Strategy Utilising Minocycline. Stroke Res Treat (2013) 2013:362961. doi: 10.1155/2013/362961 23691430 PMC3649751

[147] Jin Q Cheng J Liu Y Wu J Wang X Wei S. Improvement of Functional Recovery by Chronic Metformin Treatment Is Associated With Enhanced Alternative Activation of Microglia/Macrophages and Increased Angiogenesis and Neurogenesis Following Experimental Stroke. Brain Behav Immun (2014) 40:131–42. doi: 10.1016/j.bbi.2014.03.003 24632338

[148] Tang Z Gan Y Liu Q Yin JX Liu Q Shi J. CX3CR1 Deficiency Suppresses Activation and Neurotoxicity of Microglia/Macrophage in Experimental Ischemic Stroke. J Neuroinflammation (2014) 11:26. doi: 10.1186/1742-2094-11-26 24490760 PMC3942808

[149] Moretti R Leger PL Besson VC Csaba Z Pansiot J Di Criscio L. Sildenafil, a Cyclic GMP Phosphodiesterase Inhibitor, Induces Microglial Modulation After Focal Ischemia in the Neonatal Mouse Brain. J Neuroinflamm (2016) 13:95. doi: 10.1186/s12974-016-0560-4 PMC4850658 27126393

[150] Shu ZM Shu XD Li HQ Sun Y Shan H Sun XY. Ginkgolide B Protects Against Ischemic Stroke Via Modulating Microglia Polarization in Mice. CNS Neurosci Ther (2016) 22:729-39. doi: 10.1111/cns.12577 PMC6492915 27306494

[151] He Y Ma X Li D Hao J. Thiamet G Mediates Neuroprotection in Experimental Stroke by Modulating Microglia/Macrophage Polarization and Inhibiting NF-κB p65 signaling. J Cereb Blood Flow Metab (2017) 37:2938-51. doi: 10.1177/0271678X16679671 PMC5536801 27864466

[152] Ji J Xiang P Li T Lan L Xu X Lu G. NOSH-NBP, a Novel Nitric Oxide and Hydrogen Sulfide- Releasing Hybrid, Attenuates Ischemic Stroke-Induced Neuroinflammatory Injury by Modulating Microglia Polarization. Front Cell Neurosci (2017) 11:154. doi: 10.3389/fncel.2017.00154 28603491 PMC5445131

[153] Schmidt A Strecker JK Hucke S Bruckmann NM Herold M Mack M. Targeting Different Monocyte/Macrophage Subsets Has No Impact on Outcome in Experimental Stroke. Stroke (2017) 48:1061–9. doi: 10.1161/STROKEAHA.116.015577 28292872

[154] Jiang M Wang H Jin M Yang X Ji H Jiang. Exosomes from MiR-30d-5p-ADSCs Reverse Acute Ischemic Stroke-Induced, Autophagy-Mediated Brain Injury by Promoting M2 Microglial/Macrophage Polarization. Cell Physiol Biochem (2018) 47:864-78. doi: 10.1159/000490078 29807362

[155] Gelosa P Bonfanti E Castiglioni L Delgado-Garcia JM Gruart A Fontana L. Improvement of Fiber Connectivity and Functional Recovery After Stroke by Montelukast, an Available and Safe Anti-Asthmatic Drug. Pharmacol Res (2019) 142:223–36. doi: 10.1016/j.phrs.2019.02.025 30818044

[156] Song Y Li Z He T Qu M Jiang L Li W. M2 Microglia-Derived Exosomes Protect the Mouse Brain From Ischemia-Reperfusion Injury via Exosomal miR-124. Theranostics (2019) 9:2910-23. doi: 10.7150/thno.30879 PMC6568171 31244932

[157] Yang J Balkaya M Beltran C Heo JH Cho S. Remote Postischemic Conditioning Promotes Stroke Recovery by Shifting Circulating Monocytes to CCR2(+) Proinflammatory Subset. J Neurosci (2019) 39:7778–89. doi: 10.1523/JNEUROSCI.2699-18.2019 PMC6764204 31427395

[158] Ye Y Jin T Zhang X Zeng Z Ye B Wang J. Meisoindigo Protects Against Focal Cerebral Ischemia-Reperfusion Injury by Inhibiting NLRP3 Inflammasome Activation and Regulating Microglia/Macrophage Polarization via TLR4/NF-κB Signaling Pathway. Front Cell Neurosci (2019) 13:553. doi: 10.3389/fncel.2019.00553 31920554 PMC6930809

[159] Zheng Y He R Wang P Shi Y Zhao L Liang J. Exosomes From LPS-stimulated Macrophages Induce Neuroprotection and Functional Improvement After Ischemic Stroke by Modulating Microglial Polarization. Theranostics (2019) 7:2037-49. doi: 10.1039/c8bm01449c 30843911

[160] Li F Zhao L Shi Y Liang J. Edaravone-Loaded Macrophage-Derived Exosomes Enhance Neuroprotection in the Rat Permanent Middle Cerebral Artery Occlusion Model of Stroke. Mol Pharm (2020) 17:3192–201. doi: 10.1021/acs.molpharmaceut.0c00245 32786956

[161] Ahmed A Wang LL Abdelmaksoud S Aboelgheit A Saeed S Zhang CL. Minocycline Modulates Microglia Polarization in Ischemia-Reperfusion Model of Retinal Degeneration and Induces Neuroprotection. Sci Rep (2017) 7:14065. doi: 10.1038/s41598-017-14450-5 29070819 PMC5656679

[162] Lampl Y Boaz M Gilad R Lorberboym M Dabby R Rapoport A. Minocycline Treatment in Acute Stroke: An Open-Label, Evaluator-Blinded Study. Neurology (2007) 69:1404–10. doi: 10.1212/01.wnl.0000277487.04281.db 17909152

[163] Fagan SC Waller JL Nichols FT Edwards DJ Pettigrew LC Clark WM. Minocycline to Improve Neurologic Outcome in Stroke (MINOS): A Dose-Finding Study. Stroke (2010) 41:2283–7. doi: 10.1161/STROKEAHA.110.582601 PMC3916214 20705929

[164] Kohler E Prentice DA Bates TR Hankey GJ Claxton A van Heerden J. Intravenous Minocycline in Acute Stroke: A Randomized, Controlled Pilot Study and Meta-Analysis. Stroke (2013) 44:2493–9. doi: 10.1161/STROKEAHA.113.000780 23868273

[165] Amiri-Nikpour MR Nazarbaghi S Hamdi-Holasou M Rezaei Y. An Open-Label Evaluator-Blinded Clinical Study of Minocycline Neuroprotection in Ischemic Stroke: Gender-Dependent Effect. Acta Neurol Scand (2015) 131:45–50. doi: 10.1111/ane.12296 25155474

[166] Fouda AY Newsome AS Spellicy S Waller JL Zhi W Hess DC. Minocycline in Acute Cerebral Hemorrhage: An Early Phase Randomized Trial. Stroke (2017) 48:2885–7. doi: 10.1161/STROKEAHA.117.018658 28887388

[167] Li J Dai X Zhou L Li X Pan D. Edaravone Plays Protective Effects on LPS-Induced Microglia by Switching M1/M2 Phenotypes and Regulating NLRP3 Inflammasome Activation. Front Pharmacol (2021) 12:691773. doi: 10.3389/fphar.2021.691773 34135761 PMC8201503

[168] Enomoto M Endo A Yatsushige H Fushimi K Otomo Y. Clinical Effects of Early Edaravone Use in Acute Ischemic Stroke Patients Treated by Endovascular Reperfusion Therapy. Stroke (2019) 50:652–8. doi: 10.1161/STROKEAHA.118.023815 30741623

[169] Xu J Wang A Meng X Yalkun G Xu A Gao Z. Edaravone Dexborneol Versus Edaravone Alone for the Treatment of Acute Ischemic Stroke: A Phase III, Randomized, Double-Blind, Comparative Trial. Stroke (2021) 52:772–80. doi: 10.1161/STROKEAHA.120.031197 33588596

[170] Zhu Z Fu Y Tian D Sun N Han W Chang G. Combination of the Immune Modulator Fingolimod With Alteplase in Acute Ischemic Stroke: A Pilot Trial. Circulation (2015) 132:1104–12. doi: 10.1161/CIRCULATIONAHA.115.016371 PMC4580515 26202811

[171] Curtale G Rubino M Locati M. MicroRNAs as Molecular Switches in Macrophage Activation. Front Immunol (2019) 10:799. doi: 10.3389/fimmu.2019.00799 31057539 PMC6478758

[172] Huang S Ge X Yu J Han Z Yin Z Li Y. Increased miR-124-3p in Microglial Exosomes Following Traumatic Brain Injury Inhibits Neuronal Inflammation and Contributes to Neurite Outgrowth via Their Transfer Into Neurons. FASEB J (2018) 32:512–28. doi: 10.1096/fj.201700673R 28935818

[173] Park J Chang JY Kim JY Lee JE. Monocyte Transmodulation: The Next Novel Therapeutic Approach in Overcoming Ischemic Stroke? Front Neurol (2020) 11:578003. doi: 10.3389/fneur.2020.578003 33193029 PMC7642685

[174] Nakamura T Yamamoto E Kataoka K Yamashita T Tokutomi Y Dong YF. Pioglitazone Exerts Protective Effects Against Stroke in Stroke-Prone Spontaneously Hypertensive Rats, Independently of Blood Pressure. Stroke (2007) 38:3016–22. doi: 10.1161/STROKEAHA.107.486522 17885259

[175] Hilgendorf I Gerhardt LM Tan TC Winter C Holderried TA Chousterman BG. Ly-6chigh Monocytes Depend on Nr4a1 to Balance Both Inflammatory and Reparative Phases in the Infarcted Myocardium. Circ Res (2014) 114:1611–22. doi: 10.1161/CIRCRESAHA.114.303204 PMC4017349 24625784

[176] Etzrodt M Cortez-Retamozo V Newton A Zhao J Ng A Wildgruber M. Regulation of Monocyte Functional Heterogeneity by miR-146a and Relb. Cell Rep (2012) 1:317–24. doi: 10.1016/j.celrep.2012.02.009 PMC3334310 22545247

[177] Jackson CM Choi J Routkevitch D Pant A Saleh L Ye X. PD-1+ Monocytes Mediate Cerebral Vasospasm Following Subarachnoid Hemorrhage. Neurosurgery (2021) 88:855–63. doi: 10.1093/neuros/nyaa495 33370819

[178] Getts DR Terry RL Getts MT Deffrasnes C Muller M van Vreden C. Therapeutic Inflammatory Monocyte Modulation Using Immune-Modifying Microparticles. Sci Transl Med (2014) 6:219ra7. doi: 10.1126/scitranslmed.3007563 PMC3973033 24431111

[179] Spadaro M Montarolo F Perga S Martire S Brescia F Malucchi S. Biological Activity of Glatiramer Acetate on Treg and Anti-Inflammatory Monocytes Persists for More Than 10 Years in Responder Multiple Sclerosis Patients. Clin Immunol (2017) 181:83–8. doi: 10.1016/j.clim.2017.06.006 28642148

[180] Fischer HJ Finck TLK Pellkofer HL Reichardt HM Luhder F. Glucocorticoid Therapy of Multiple Sclerosis Patients Induces Anti-Inflammatory Polarization and Increased Chemotaxis of Monocytes. Front Immunol (2019) 10:1200. doi: 10.3389/fimmu.2019.01200 31191554 PMC6549240

[181] Han R Xiao J Zhai H Hao J. Dimethyl Fumarate Attenuates Experimental Autoimmune Neuritis Through the Nuclear Factor Erythroid-Derived 2-Related Factor 2/Hemoxygenase-1 Pathway by Altering the Balance of M1/M2 Macrophages. J Neuroinflamm (2016) 13:97. doi: 10.1186/s12974-016-0559-x PMC4855950 27142843

[182] Degan D Ornello R Tiseo C Carolei A Sacco S Pistoia F. The Role of Inflammation in Neurological Disorders. Curr Pharm Des (2018) 24:1485–501. doi: 10.2174/1381612824666180327170632 29589534

[183] Lassmann H. Pathology of Inflammatory Diseases of the Nervous System: Human Disease Versus Animal Models. Glia (2020) 68:830–44. doi: 10.1002/glia.23726 PMC7065008 31605512

